# The interaction of salinity and light regime modulates photosynthetic pigment content in edible halophytes in greenhouse and indoor farming

**DOI:** 10.3389/fpls.2023.1105162

**Published:** 2023-04-04

**Authors:** Maria Fitzner, Monika Schreiner, Susanne Baldermann

**Affiliations:** ^1^ Department Plant Quality and Food Security, Leibniz Institute of Vegetable and Ornamental Crops (IGZ), Grossbeeren, Germany; ^2^ Food Chemistry, Institute of Nutritional Science, University of Potsdam, Nuthetal, Germany; ^3^ Food4Future (F4F), c/o Leibniz Institute of Vegetable and Ornamental Crops (IGZ), Department Plant Quality and Food Security, Grossbeeren, Germany; ^4^ Food Metabolome, Faculty of Life Science: Food, Nutrition and Health, University of Bayreuth, Kulmbach, Germany

**Keywords:** saline agriculture, vertical farming, abiotic stress, vegetables, future food production, light condition

## Abstract

Given its limited land and water use and the changing climate conditions, indoor farming of halophytes has a high potential to contribute significantly to global agriculture in the future. Notably, indoor farming and classical greenhouse cultivation differ in their light regime between artificial and solar lighting, which can influence plant metabolism, but how this affects the cultivation of halophytes has not yet been investigated. To address this question, we studied the yield and content of abscisic acid, carotenoids, and chlorophylls as well as chloride of three halophyte species (*Cochlearia officinalis*, *Atriplex hortensis*, and *Salicornia europaea*) differing in their salt tolerance mechanisms and following four salt treatments (no salt to 600 mM of NaCl) in two light regimes (greenhouse/indoor farming). In particular, salt treatment had a strong influence on chloride accumulation which is only slightly modified by the light regime. Moreover, fresh and dry mass was influenced by the light regime and salinity. Pigments exhibited different responses to salt treatment and light regime, reflecting their differing functions in the photosynthetic apparatus. We conclude that the interaction of light regime and salt treatment modulates the content of photosynthetic pigments. Our study highlights the potential applications of the cultivation of halophytes for indoor farming and underlines that it is a promising production system, which provides food alternatives for future diets.

## Graphical Abstract

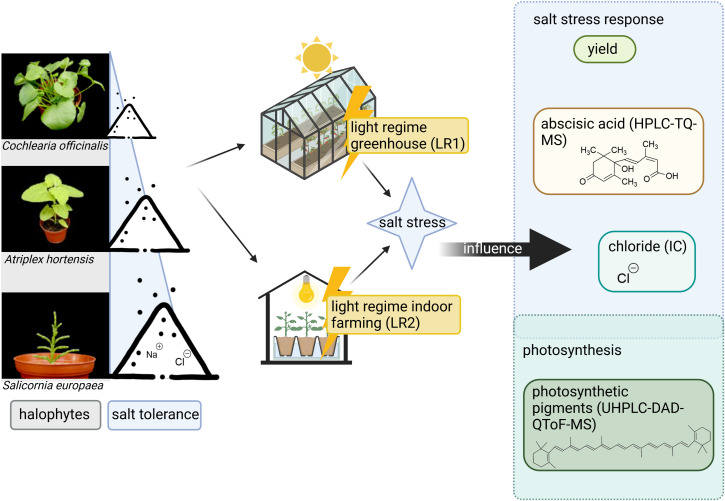

## Introduction

Reducing land use and water consumption is among the biggest challenges for future food production (FAO, 2020). One approach to overcome these challenges is to combine indoor farming with saline agriculture. Indoor farming holds the capability for year-round uniform product quantity and quality due to controlled environmental conditions. These systems are efficient in resource use through smart climate systems, such as heating, ventilation, and air conditioning (HVAC); fertigation systems, such as the nutrient film technique (NFT); and the use of innovative sensing, modeling, and AI technologies (Asseng et al., 2020; van Delden et al., 2021; Swain, 2022). Saline agriculture offers the possibility to use saline water resources and, thus, minimize the freshwater use of the system to zero (Norton, 2021). Crucially, saline indoor farming could open new opportunities for the exploitation of the vast untapped potential of not only saline water sources but also unused urban areas thereby revolutionizing sustainable agricultural practices and reducing food miles (Norton, 2021). Salt plants (halophytes) are potential candidates for saline indoor farming since they tolerate high salinity levels and still contribute to a healthy diet, due to their content of plant secondary metabolites (PSMs). Nevertheless, the quantity and quality of these alternative vegetables depend on environmental conditions, and thus, it is important to investigate optimal growth conditions for new indoor farming systems.

This study is focused on three different edible halophytes: scurvy grass (*Cochlearia officinalis*), garden orache (*Atriplex hortensis*), and glasswort (*Salicornia europaea*). Halophytes are plants that can grow and reproduce under saline conditions. A key aspect of salinity tolerance of halophytes is the osmotic adjustment, which is different from glycophytes (non-halophytic plants), and also differs among halophyte species (Flowers and Colmer, 2008). Halophytes that tolerate salt, but do not require salt for growth, and therefore grow optimally in non-saline to moderately saline environments, are classified as facultative halophytes (Hasanuzzaman et al., 2014). There are also halophyte species, which require salt for optimal growth and are classified as obligate halophytes (Hasanuzzaman et al., 2014). While *A. hortensis* and *C. officinalis* show optimal growth at no salt or medium salinity (up to 100 mM), *S. europaea* shows optimal growth between 200 and 400 mM of salt (Wilson et al., 2000; Kachout et al., 2009; Lv et al., 2012; de Vos and Broekman, 2013; He et al., 2017). To adapt to salinity, halophytes have evolved different salt tolerance mechanisms, namely, the salt-excluding, salt-excreting, and salt-accumulating mechanisms. The salt-excluding mechanism reduces salt uptake by the roots, the salt-excreting mechanism eliminates salt through salt bladders/glands, and the salt-accumulating mechanism promotes the storage of salt in cell vacuoles (Hasanuzzaman et al., 2014; Chen et al., 2018). *Salicornia europaea* can be assumed to be a salt-accumulating plant since it develops stem succulence (Song and Wang, 2014; Araus et al., 2021). Most *Atriplex* spp., including *A. hortensis*, are salt-excreting plants and form salt bladders (Schirmer and Breckle, 1982; Breckle, 2002; Kachout et al., 2009; Yuan et al., 2016). de Vos and Broekman (2013) classifies *C. officinalis* as an intermediate halophyte but neither as a salt-accumulating plant nor as a plant-forming salt bladder/gland. It can be assumed that *C. officinalis* is a salt-excluding plant even though detailed studies are missing.

As with glycophytes, salt stress occurs in halophytes above the tolerable salt levels, although these levels vary among halophyte species. Salt stress can be indicated by signaling molecules, such as the plant hormone abscisic acid (ABA). ABA is known to be an essential signaling molecule and regulatory factor in response to salt stress (Zhu, 2002; Golldack et al., 2014). An important effect of ABA is to induce the closure of stomata by guard cells (Tuteja, 2007). This mechanism is essential for plants’ water status and involves the sensing of air humidity by the guard cells and water potential by the roots (Julkowska and Testerink, 2015; Ko and Helariutta, 2017). Recent studies suggest differential regulation of ABA metabolism in halophytic and glycophytic guard cells (Ellouzi et al., 2014; Karimi et al., 2021). However, only a few studies have considered both ABA and the salt tolerance mechanism. For example, Ben Hassine et al. (2009) indicate that ABA may be involved in the regulation of salt excretion in *Atriplex halimus*.

There has been a dramatic increase in halophyte research in recent years, including halophyte agriculture for food production (Abdelly et al., 2022). Although many studies focus on halophyte agriculture in the greenhouse or field (Ladeiro, 2012; de Vos and Broekman, 2013; Ventura and Sagi, 2013), very few studies focus on the indoor farming of halophytes (Norton, 2021). A central advantage of indoor farming is that the environmental conditions can be modulated to a full extent. In contrast, greenhouse cultivation still depends on outdoor environmental conditions. For example, there is a great difference in the daily light integral (DLI) in greenhouse cultivation, which is dependent on solar lighting, during the year. Especially in winter months (November–February) in the northern latitudes, the DLI is significantly lower than in summer (Korczynski et al., 2002; Hernandez Velasco, 2021). These indifferences in lighting can lead to inconsistent quality of crops. Artificial lighting in indoor farming, on the other hand, offers a year-round uniform product and the possibility to optimize lighting conditions for yield and nutritional profile. Still, aside from the DLI, the differences between artificial and solar lighting (natural light) also include differences in spectral quality and diurnal changes, which also can affect crop yield and nutritional quality. For instance, Annunziata et al. (2017) grew *Arabidopsis thaliana* plants under natural light and two artificial light sources (fluorescent and LED light), whereby the different light sources resulted in changes in plant metabolism, such as diurnal changes in carbohydrate or amino acid metabolism, which are dependent on the light source.

Similarly, PSMs are influenced by light conditions. Several studies have investigated the influence of light conditions on the composition of PSMs in glycophytes (non-halophytic plants), such as different colored light-emitting diodes (LEDs) or ultraviolet (UV) radiation (Heinze et al., 2018; Naznin et al., 2019; Maina et al., 2021). Notably, the influence of light qualities on PSMs is both species-specific and metabolite-specific. Carotenoids and chlorophylls are of particular interest due to their function as photosynthetically active pigments and their photoprotective properties associated with changing light conditions. Chlorophyll *a* is a light-harvesting molecule that converts light energy into chemical energy (Björn et al., 2009). Chlorophyll *b* is important for the stabilization of the light-harvesting complex (LHC) (Tanaka and Tanaka, 2011). Carotenoids act not only as light harvesters but also as scavengers of reactive oxygen species (ROS). For example, β-carotene protects photosystem II (PSII) from photooxidative damage by quenching singlet oxygen formed in PSII (Choudhury and Behera, 2001; Hideg et al., 2002; Trebst, 2003). Zeaxanthin is known to be involved in non-photochemical quenching (NPQ), which plants use to dissipate excess light energy (Gilmore, 2001). Violaxanthin plays a very important role in plants in dissipation in case the light exceeds the uptake capacity of the photochemical apparatus (photoprotection) (Gilmore, 2001). Since chlorophylls and carotenoids have individual functions in plants, it is important to evaluate them individually. Furthermore, carotenoid metabolism is not only affected by light (Lado et al., 2015; Frede et al., 2018; Frede et al., 2019; Frede and Baldermann, 2022) but also by salinity (Kim et al., 2008). Since salinity-induced changes in PSM levels in halophytes are observed (Aghaleh, 2011), it is likely that the interaction between salinity and light conditions also affects PSMs, as salt stress also influences photosynthesis.

The effect on PSMs is of particular interest because of their health-promoting effects when consumed in the human diet. In particular, carotenoids are crucial components of the human diet as they have been associated with the prevention of non-communicable diseases such as cancer and diabetes. This is attributed to their chemoprotective properties (Fiedor and Burda, 2014). Halophytes, for example, *S. europaea*, exhibit a rich profile of secondary metabolites (Kim et al., 2021).

Indeed, investigating the impact of environmental conditions on yield and PSMs is a key issue for food produced in indoor farming. To address this, our study was designed to compare the light regimes of greenhouse and indoor farming and their effects on salt stress response and photosynthetic pigments in halophytes. Considering this, we evaluated the effect of salt treatment on yield, chloride accumulation, and ABA content as well as individual carotenoids and chlorophylls in the leaves of three different halophyte species (*C. officinalis*, *A. hortensis*, and *S. europaea*) grown in two different light regimes (greenhouse and indoor farming).

The study demonstrates that halophytes adapt species-specific to changing light and salt environments and that these factors mutually influence each other.

## Materials and methods

### Plant material and cultivation

The seeds of *S. europaea* were purchased from Rühlemann’s Kräuter & Duftpflanzen (Germany) and the seeds of *C. officinalis* and *A. hortensis* were from Magic Garden Seeds (Germany). The plants were germinated in soil [substrate type P; pH = 5.9; 120 mg L^−1^ of N; 120 mg L^−1^ of ${\text{PO}}_{4}^{2-}$ ; 170 mg L^−1^ of K; 120 mg L^−1^ of Mg; density, 430 kg/m^3^; 70% raised bog peat (degree of decomposition: H2-H5), 30% clay; Einheitserdewerke Werkverband e.V., Germany]. When two leaves had fully developed, the plants were transferred to pots (diameter, 8 cm) containing one-third of soil [substrate type T; pH 5.9; 180 mg L^−1^ of N; 180 mg L^−1^ of ${\text{PO}}_{4}^{2-}$ ; 260 mg L^−1^ of K; 130 mg L^−1^ of Mg; density, 430 kg/m^3^; 70% raised bog peat (degree of decomposition: H2-H5), 30% clay; Einheitserdewerke Werkverband e.V., Germany], one-third of fine quartz sand (grain size 0.5–1 mm), and one-third of coarse quartz sand (grain size 2–3 mm) (Euroquarz GmbH, Germany). The water content of the soil with respect to salt treatment can be found in **Table S2**. The plants were irrigated with a modified Hoagland solution (**Table S1**).

### Light regimes and salt treatment

#### Light regimes

The greenhouse cultivation, light regime 1 (LR1), was located at the Leibniz Institute of Vegetable and Ornamental Crops (Grossbeeren, 52°20′5N, 13°18′35.3′′E), and the experiment was conducted in November 2019. The lighting setup consisted of natural light and an additional artificial light source (SON-T Agro 400W; Philips, The Netherlands) for 7 h per day from 05:00 to 12:00 o’clock. Thus, on average, the plants were grown under a light–dark regime of 11 h of light and 13 h of darkness. The intensity of natural light was measured in photosynthetic photon flux density (PPFD) using a PAR sensor (LI-190R Quantum Sensor, LICOR Biosciences GmbH, Germany) on the roof of the greenhouse and calculated based on the light transmittance of the glass (50%). Based on these data, the daily light hours, intensity, and DLI were calculated, taking into account natural and artificial light (**Table 1**). Since the two replicate experiments were performed simultaneously, their lighting conditions were the same.

**Table 1:** Light settings of light regime 1 (LR1, greenhouse) and light regime 2 (LR2, indoor farming) (means ± SEM).

		Light hours (h day^−1^)	Light intensity (PPFD) (µmol m^−2^ s^−1^)	Daily light integral (DLI) (mol m^−2^ day^−1^)		
				Average	Min	Max
Light regime 1	Artificial light	7	46.93 ± 1.32			
	Natural light^a^	8.61 ± 0.04	49.47 ± 4.45			
	Combined light sources^b^	11.06 ± 0.04	66.54 ± 6.84	3.18 ± 0.26	1.86 ± 0.14	4.33 ± 0.4
Light regime 2	Climate chamber 1	14	366.23 ± 4.60	18.46 ± 0.23		
	Climate chamber 2		346.93 ± 4.70	17.49 ± 0.24		
	Average^c^		356.58 ± 4.7	17.98 ± 0.24		

PPFD, photosynthetic photon flux density.

a Average values calculated for the time period of the experiment (17 days) in light regime 1 with data taken from a sensor on top of the greenhouse.

b Values were calculated by including artificial and natural light.

c Values were calculated by the average of both climate chambers.

The indoor farming system, light regime 2 (LR2), was set up in a climate chamber (Vötsch Industrietechnik GmbH, Germany), also in November 2019. The lighting setup consisted only of artificial lighting (Clean Ace™ R MT400DL/BH YE; EYE Lighting Europe Ltd., United Kingdom), where the plants were grown under a light–dark regime of 14 h of light and 10 h of darkness (**Table 1**). Replicate experiments were conducted in identical climate chambers at the same time, but light intensity (PPFD) differs slightly and is given for climate chambers 1 and 2 (**Table 1**).

The remaining adjustable climatic conditions were set the same in both greenhouse and indoor farming: temperature, 22°C/18°C (day/night); humidity, 65%; and CO_2_, 400 ppm.

#### Salt treatment

The plants were irrigated in an NFT system in 0.5-h intervals (**Table S1**). Plants were acclimated to the NFT system for 1 week prior to salt treatment in both light regimes. Four salt concentrations, no salt, or 50, 200, or 600 mM of sodium chloride, were utilized to study the effect of salt in the NFT system. Salt treatment was initiated by adding the desired salt concentrations to the nutrient solution in a single step. This time point is considered the start of the experiment, and the salt concentrations were monitored from then onwards and adjusted as necessary throughout the experimental period (**Figure S4**). After 17 days of treatment, the plants were harvested. The chloride content in the substrate was determined at the end of the experiment (**Table S2**).

### Plant sampling and fresh and dry mass

To determine the fresh mass, the 12 plants were cut at the root and then the aboveground part of the plants was weighed as whole plants; then, three plants were pooled and the pooled leaves were weighed separately. For further analysis of the metabolite content, the main leaves of *A. hortensis* and *C. officinalis* were harvested, and the green aboveground part (which is later on referred to as leaves) of *S. europaea* was harvested and pooled into four technical replicates per salt treatment at each experiment, resulting in eight replicates per light regime from two independent experiments. After harvesting, the plants were immediately frozen in liquid nitrogen and then freeze-dried for 1 week until completely dry. Dry mass was determined by weighing the pooled leaves before (FM) and after (DM) freeze drying. Percent dry mass was calculated as DM/FM*100. For further analysis, plant samples were homogenized using a Retsch mill (Retsch MM 400; Retsch GmbH, Germany) [three to five times for 50 s with three to five metal beads (diameter, 9 mm) at 25 Hz].

### Determination of chloride concentration in the leaves

The chloride content of the sampled leaves was determined by ion chromatography. For this purpose, 10 mg of dried plant material was dissolved in 1 ml of ultrapure water. As an internal standard, 0.5 ml of sodium bromide solution (0.6 mg ml^−1^) was added. The samples were sonicated on ice for 10 min and then centrifuged for 5 min (4,500×*g,* 4°C). Next, the samples were diluted with ultrapure water, according to the expected salt concentration of the samples, to fit into the calibration range of chloride. Chloride determination was carried out using a 930 Compact IC Flex ion chromatograph (Metrohm AG, Switzerland) equipped with a conductivity detector and suppression system. A Metrosep A Supp 5-250/4.0 column was used with a flow rate of 0.7 ml min^−1^ and an injection volume of 20 µl. Gradient elution was performed using Na_2_CO_3_ (3.2 mM) and NaHCO_3_ (1 mM). The final chloride concentration was calculated with external calibration using a chloride standard (>99%; Carl Roth GmbH, Germany).

### Determination of ABA content in the leaves

Determination of ABA content was performed as previously described (Errard et al., 2015) with modifications. In brief, 10 mg of the dried plant material was extracted with 0.2 ml of methanol/water (60:40, v/v), and an internal standard [(+)-abscisic acid-d6, Toronto Research Chemicals, Canada] was added. First, the solution was sonicated on ice for 15 min and then centrifuged for 10 min (12,298×g, 4°C). Next, the supernatant was collected in a micro reaction vessel and the extraction steps were repeated twice. Then, the collected supernatant was filtered through a PTFE filter tube (0.2 µm, Thermo Fisher Scientific Inc., USA) and transferred to HPLC vials. Finally, the filtrate was diluted 1:2 with MS water (Supelco, VWR, Germany) + 0.1% acetic acid. The measurement was performed using an Agilent Technologies 1260 Infinity HPLC (Agilent Technologies Sales and Services GmbH & Co. KG, Germany) in combination with a Triple Quadrupole Q-Trap^®^ 6500-MS/MS system (AB Sciex LLC, USA). Chromatographic separation was performed using a Zorbax Eclipse Plus C18 column (1.8 µm, 2.1 mm × 50 mm; Agilent Technologies, Germany), a column temperature of 30°C, a flow rate of 650 µl min^−1^, and a mobile phase consisting of solvent A: MS water + 0.1% acetic acid and solvent B: acetonitrile + 0.1% ultrapure water. The injection volume was 10 µl. The initial gradient was 90% solvent A for 1 min, reduced to 15% solvent A for 4 min, and then reduced to 0% solvent A for 4 min. The mass spectrometer was operated in negative ionization mode and an electron spray ionization source was used. The MS parameters were set as follows: ion source temperature, 500°C; ion spray voltage, −4,500 V; curtain gas pressure, 50 psi; drying gas pressure, 50 psi; nebulizer gas pressure, 50 psi; auxiliary gas pressure, 65 psi; and multireaction monitoring (MRM) at a dwell time of 0.3781 s. Identification was based on the retention time and MRM transitions of the following: ABA 263 → 153 [quantifier; collision energy (CE), −15 V], 263 → 203 (qualifier; CE, −40 V), and 263 → 122 (qualifier; CE, −48 V) and ABA-d6 269 → 159 (quantifier; CE, −15 V), 269 → 209 (qualifier; CE, −40 V), and 269 → 128 (qualifier; CE, −48 V). The final ABA concentration was calculated from a calibration curve of the quantifier ratios between an ABA standard (≥98.5%, Sigma Aldrich) and the internal standard. Data analysis was performed with the Analyst 1.6.2 software (AB Sciex LLC, USA).

### Identification and quantification of chlorophylls and carotenoids in the leaves

The extraction of pigments was performed according to Frede et al. (2019). In brief, 5 mg of plant material was dissolved in 0.5 ml of methanol/tetrahydrofuran (1:1, v/v) and incubated for 5 min in a shaker (1,400 rpm, 20°C) followed by centrifugation for 5 min (4,500×g, 20°C). The supernatant was collected in a vial, and the extraction was performed five times. The solution was evaporated to dryness under a nitrogen stream, dissolved in dichloromethane/isopropanol (1:5, v/v), sonicated (3 min, 20°C), filtered (PTFE filter tubes), and transferred to an HPLC vial. The analysis was performed using Agilent Technologies 6530 QToF-DAD-UHPLC-MS (Agilent Technologies Sales and Services GmbH & Co. KG, Germany) according to Frede et al. (2017). Identification was achieved using mass spectra and UV/VIS spectra (**Figure S1**), and quantification was achieved using external calibration with carotenoid standards (CaroteNature GmbH, Switzerland) of *all-trans*-isomers from β-carotene, lutein, and zeaxanthin and 9-*cis*-neoxanthin as well as chlorophyll standards (Sigma Aldrich Chemie GmbH, Germany) of chlorophyll *a* and *b* at a wavelength of 450 nm. Data analysis was performed using the TOF Quantitative Analysis (Quant-My-Way) 10.2 (MassHunter, USA).

### Statistical analysis

Statistical differences between the light regime and salt treatments were tested using SigmaPlot (14.0) with a two-way ANOVA followed by a Bonferroni *post-hoc* test (*p* ≤ 0.05) (*dF*, *F*, and *p*-values represented in **Table S3**). Data are presented as means ± SEM of two individual experiments per light regime. Twenty-four plants per light regime were used for the determination of fresh mass. For the analysis of the selected metabolites, eight replicates per light regime were used, pooled from three individual plants and two independent experiments.

## Results

### Characterization of light regimes

To evaluate the variation in both light regimes, LR1 and LR2, the light spectra, light intensity, and daily light hours were measured (**Table 1**; **Figures S2, S3**). The major differences between both light regimes were detected in the daily light hours and light intensity. To encounter both, the DLI was calculated (**Table 1**). Light regime 1 showed an average DLI of 3.18 ± 0.26 µmol m^−2^ day^−1^ that was only 18% of the DLI of light regime 2, which was on average 17.98 ± 0.24 µmol m^−2^ day^−1^. This is due to 3 h less day light and a 290 µmol m^−2^ s^−1^ lower light intensity in light regime 1 (LR1, greenhouse) compared with light regime 2 (LR2, indoor farming). Light regime 1 showed, due to variations in natural light, variations in DLI (**Figure S2**).

### Identifying differences in salt stress response between light regimes

#### Effect of salt treatment and light regime on fresh and dry mass

**Figure 1:**
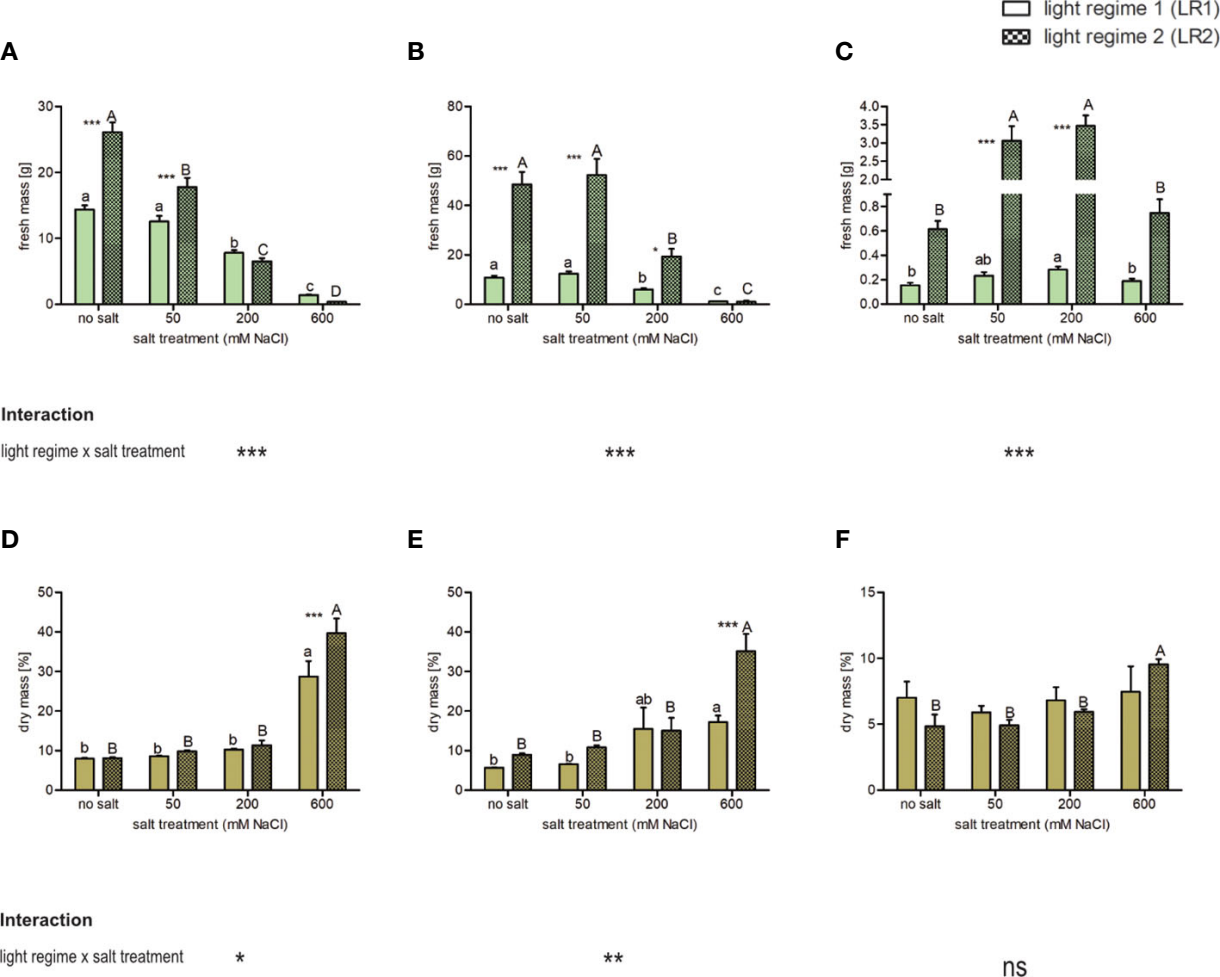
Influence of salt treatment and light regime on the fresh mass of plants and percent dry mass (as a percentage of fresh mass) of the leaves of 6- to 9-week-old plants. Fresh mass **(A–C)** and dry mass **(D–F)** of **(A, D)** *Cochlearia officinalis*, **(B, E)** *Atriplex hortensis*, and **(C, F)** *Salicornia europaea*. Fresh mass: the bar represents means ± SEM, *n* = 24; dry mass: the bar represents means ± SEM of *n* = 8 pools of three individual plants each from two independent experiments. Small letters indicate significant differences between salt treatments in light regime 1 (LR1, greenhouse) in alphabetical order from highest to lowest; capital letters indicate significant differences between salt treatments in light regime 2 (LR2, indoor farming) in alphabetical order from highest to lowest; asterisks indicate significant differences between LR1 and LR2 in between one salt treatment; interaction shows significantly different interactions between salt treatments and light regimes tested by two-way ANOVA, followed by *post-hoc* Bonferroni test (*p* ≤ 0.05) (*≤ 0.05, **≤ 0.01, ***≤ 0.001); ns, not significant.

To assess whether fresh and dry mass is affected by the salt treatment and whether this response depends on the light regime, we measured the fresh mass of the plants during harvest and determined the dry mass of the leaves after lyophilization. The percent dry mass represents the proportion of dry mass in fresh mass and thus increases with decreasing water content. We found that fresh and dry mass was affected by both the light regime and salt treatment (**Figure 1**; **Table S4**). Considering the plant response to salt treatment, we found that *C. officinalis* showed a salt-induced decrease in fresh mass in both light regimes. Considering the plant response to the light regime, this decrease is in LR2 (indoor farming) beginning from 50 mM of salt and in LR1 (greenhouse) from 200 mM (**Figure 1A**). Accordingly, the highest percent dry mass was found at 600 mM of salt, which was due to the lowest water content in both light regimes (**Figure 1D**). Similarly, in *A. hortensis*, we observed a decrease in fresh mass, but only at salt treatments greater than 200 mM in both light regimes (**Figure 1B**). The percent dry mass was also the highest at 600 mM of salt and higher in LR2 (indoor farming) (**Figure 1E**). *Salicornia europaea* showed an increased fresh mass at 50 and 200 mM of salt in LR2 (indoor farming) and at 200 mM of salt in LR1 (greenhouse) (**Figure 1C**). The percent dry mass was significantly different only in LR2 (indoor farming) and was also the highest within the 600 mM salt treatment group (**Figure 1F**). In contrast to the other two halophyte species, the percent dry weight and thus the water content in *S. europaea* changed only by approximately 5%, whereas in *C. officinalis* and *A. hortensis*, these were changed by approximately 30%. We also observed that the plants had a 0.8-fold (*C. officinalis*) to 3.5-fold (*A. hortensis*) higher fresh mass in LR2 (indoor farming) than plants grown in LR1 (greenhouse). The plants showed an interaction between salt treatment and light regime, expressed in a higher fresh mass in LR2 (indoor farming) in their salt tolerance range, and there were no differences in the salt stress range.

In summary, we observed in the salt tolerance range a significant difference in yield between the two light regimes, but in the salt stress range, the light regime had no effect on yield (except for *A. hortensis* at 200 mM, specific salt tolerance/stress ranges are defined in Section 4.1). *Vice versa*, there was a difference in percent dry mass at the highest salt level (600 mM) between both light regimes and compared with lower salt treatments.

#### Differences in chloride accumulation in the leaves

**Figure 2:**
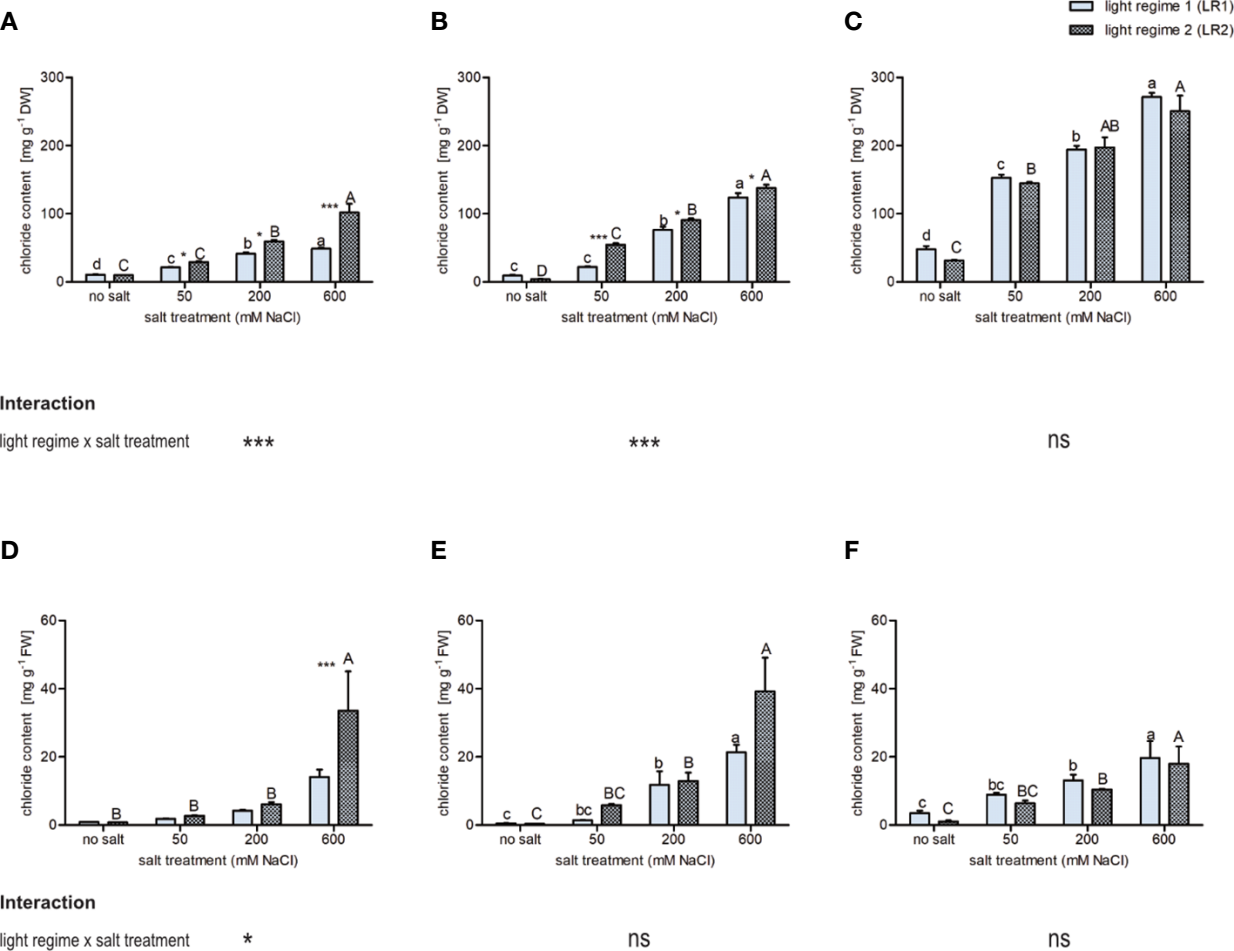
Influence of salt treatment and light regime on chloride concentration on **(A–C)** dry mass basis and **(D–F)** fresh mass basis in the leaves of 6- to 9-week-old plants. **(A, D)** *Cochlearia officinalis*; **(B, E)** *Atriplex hortensis*; **(C, F)** *Salicornia europaea*. Bar represents means ± SEM of *n* = 8 pools of three individual plants each from two independent experiments. Small letters indicate significant differences between salt treatments in light regime 1 (LR1, greenhouse) in alphabetical order from highest to lowest; capital letters indicate significant differences between salt treatments in light regime 2 (LR2, indoor farming) in alphabetical order from highest to lowest; asterisks indicate significant differences between LR1 and LR2 in between one salt treatment; interaction shows significantly different interactions between salt treatments and light regimes tested by two-way ANOVA, followed by *post-hoc* Bonferroni test (*p* ≤ 0.05) (* ≤ 0.05, *** ≤ 0.001); ns, not significant.

To evaluate whether the light regime influences chloride accumulation in the leaves, the chloride concentrations were determined *via* ion chromatography. Since salt affects water uptake and thus water content, chloride content is shown on a dry and fresh mass basis (**Figure 2**). In all three plant species, chloride concentration was slightly influenced by the light regime and highly influenced by the salt treatment. On a dry mass basis, *C. officinalis* and *S. europaea* showed the lowest and the highest chloride concentrations, respectively, in all salt treatments and in both light regimes. We observed for all plant species a positive correlation between chloride concentration and salt treatment. Comparing the two light regimes, *C. officinalis* showed slightly higher chloride accumulation at 50 and 200 mM of salt and significantly higher chloride accumulation at 600 mM of salt in LR2 (indoor farming). This finding is consistent with the necrotic phenotype of the plants observed at 600 mM of salt in LR2 (indoor farming) (**Figure 2A** and **Figure S5**). In contrast, *A. hortensis* showed significantly higher chloride concentration at 50 mM of salt and slightly higher chloride concentration at 200 and 600 mM of salt in LR2 (indoor farming) (**Figure 2B**). For *A. hortensis*, we observed salt deposition on the leaf and stem surfaces (**Figure S6**). Interestingly, *S. europaea* showed the same response under both light regimes (**Figure 2C**). Chloride content on a fresh mass basis showed the same pattern with respect to salt treatment, but with less significant changes (**Figures 2D–F**). For example, in *A. hortensis*, no significant differences were observed with respect to the light regime, but the contents tended to be higher in LR2 (indoor farming). When comparing the plant species at 600 mM, the chloride content decreased from *A. hortensis* through *C. officinalis* to *S. europaea*. *Salicornia europaea* showed a four to eight times lower increase at 200 and 600 mM of salt compared with no salt and to the other halophytic plant species.

Taken together, these findings indicate that chloride accumulation was less influenced by the light regime than by salt treatment. However, we observed an interaction between the light regime and salt for all treatments for both *C. officinalis* and *A. hortensis*.

#### Response to the light regime and salt treatment on ABA content

**Figure 3:**
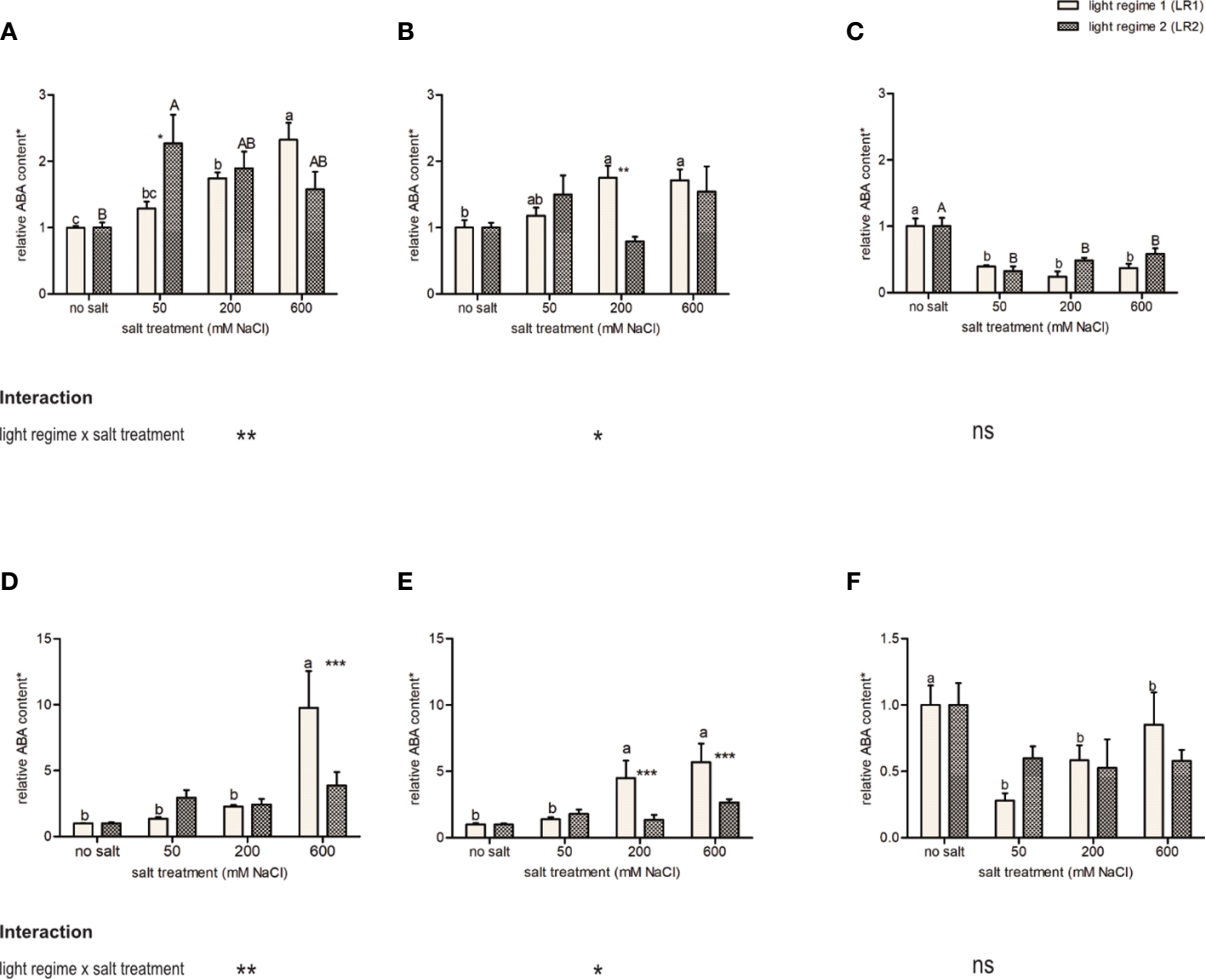
Influence of salt treatment and light regime on abscisic acid (ABA) content on **(A–C)** dry mass basis and **(D–F)** fresh mass basis in the leaves of 6- to 9-week-old plants. **(A, D)** *Cochlearia officinalis*; **(B, E)** *Atriplex hortensis*; **(C, F)** *Salicornia europaea*. *Relative data of the respective control of light regime; bar represents means ± SEM of *n* = 8 pools of three individual plants each from two independent experiments. Small letters indicate significant differences between salt treatments in light regime 1 (LR1, greenhouse) in alphabetical order from highest to lowest; capital letters indicate significant differences between salt treatments in light regime 2 (LR2, indoor farming) in alphabetical order from highest to lowest; asterisks indicate significant differences between LR1 and LR2 in between one salt treatment; interaction shows significantly different interactions between salt treatments and light regimes tested by two-way ANOVA, followed by *post-hoc* Bonferroni test (*p* ≤ 0.05) (* ≤ 0.05, ** ≤ 0.01, *** ≤ 0.001); ns, not significant.

As an indicator of salt stress in plants, ABA content in the leaves was measured using HPLC-MS/MS. Considering ABA content, we observed a different response to salt treatment as well as light regimes between obligate and facultative halophytes (**Figure 3**). The facultative halophytes *C. officinalis* and *A. hortensis* showed a positive correlation between ABA content and salinity but responded differently to salt treatment in LR1 (greenhouse) than in LR2 (indoor farming). *Cochlearia officinalis* showed the highest ABA content at 50 mM of salt in LR2 (indoor farming) and at 600 mM of salt in LR1 (greenhouse) (**Figure 3A**). *Atriplex hortensis* showed no significant differences in ABA content in LR2 (indoor farming) but showed increased ABA content at 200 and 600 mM of salt compared with no salt in LR1 (greenhouse) (**Figure 3B**). The obligate halophyte *S. europaea* showed decreased ABA content in the salt treatments compared with the no-salt treatment but showed no changes in ABA content related to light regimes (**Figure 3C**). On fresh mass, we observed the same trend, but with no significant changes in LR2 (indoor farming) for all plant species (**Figures 3D–F**). However, *C. officinalis* was found to have significantly higher ABA content at 600 mM of salt compared with the other treatments in LR1 (greenhouse) as well as *A. hortensis* at 200 and 600 mM of salt at LR1 (greenhouse) compared with the 50 mM and no salt.

The facultative halophytes (*C. officinalis* and *A. hortensis*) showed an interaction of salt and light regime (**Figure 3**) and a different response to salt treatment in the light regimes, while the obligate halophyte (*S. europaea*) showed no interaction and no difference in response to the light regimes.

### Influence of light regime and salt treatment on photosynthetic pigment content

To estimate the effect of the light regime on salt treatment on photosynthesis, we analyzed the pigment (carotenoids and chlorophylls) content by UHPLC-DAD-QToF-MS. Chlorophyll *a* and *b*, as well as *all-trans*-isomers of lutein, β-carotene, zeaxanthin, and violaxanthin and the 9-*cis*-isomer of neoxanthin, were detected in all plant species (**Figure S1**). Both carotenoids and chlorophylls were affected by salt treatment and light regime, and an interaction between these two factors was observed, with different responses for facultative halophytes (*C. officinalis* and *A. hortensis*) and the obligate halophyte (*S. europaea*). Due to the high impact on water content, the metabolites are presented on a dry mass basis (**Tables 2**, **3**), while the results based on a fresh mass basis can be found in **Tables S5, S6**.

#### Chlorophyll content in the leaves

Halophytes responded differently to salt treatment in the two light regimes, and both regimes affected chlorophyll content (**Table 2**, dry mass basis; **Table S5**, fresh mass basis).

**Table 2:** Content of chlorophylls on a dry mass basis in the leaves of 6- to 9-week-old plants (means ± SEM).

		Salt treatment (mM NaCl)	Chlorophyll *a* (µg mg^−1^ DM)					Chlorophyll *b* (µg mg^−1^ DM)				
*Cochlearia officinalis*	Light regime 1	No salt	8.59	±	0.50	a	**	2.81	±	0.15	a	***
		50	8.90	±	0.13	a	***	2.94	±	0.07	a	***
		200	8.25	±	0.18	a	***	2.77	±	0.05	a	***
		600	6.05	±	0.22	b	***	2.17	±	0.09	b	***
	Light regime 2	No salt	7.26	±	0.20	A		2.32	±	0.07	A	
		50	6.65	±	0.10	A		2.13	±	0.05	A	
		200	4.72	±	0.07	B		1.56	±	0.04	B	
		600	1.47	±	0.22	C		0.54	±	0.08	C	
Interaction: light regime × salt treatment							***					***
*Atriplex hortensis*	Light regime 1	No salt	3.15	±	0.11	a		0.72	±	0.01	a	
		50	3.31	±	0.07	a		0.69	±	0.02	a	
		200	3.14	±	0.06	a	***	0.67	±	0.01	a	***
		600	1.74	±	0.10	b	***	0.41	±	0.04	b	***
	Light regime 2	No salt	3.82	±	0.12	A	***	0.75	±	0.03	A	
		50	3.34	±	0.05	B		0.62	±	0.02	B	
		200	2.50	±	0.15	C		0.49	±	0.03	C	
		600	0.49	±	0.10	D		0.10	±	0.02	D	
Interaction: light regime × salt treatment							***					***
*Salicornia europaea*	Light regime 1	No salt	0.54	±	0.24	b		0.51	±	0.15	ns	
		50	0.78	±	0.27	ab		0.50	±	0.06	ns	
		200	1.49	±	0.17	a		0.57	±	0.05	ns	
		600	0.91	±	0.07	ab		0.37	±	0.04	ns	
	Light regime 2	No salt	0.62	±	0.20	C		0.44	±	0.07	C	
		50	3.21	±	0.20	A	***	1.01	±	0.04	A	***
		200	2.67	±	0.11	A	***	0.79	±	0.04	B	
		600	1.74	±	0.07	B	**	0.55	±	0.02	C	
Interaction: light regime × salt treatment							***					**

Small letters indicate significant differences between salt treatments in light regime 1 (LR1, greenhouse) in alphabetical order from highest to lowest; capital letters indicate significant differences between salt treatments in light regime 2 (LR2, indoor farming) in alphabetical order from highest to lowest; asterisks indicate significant differences between LR1 and LR2 in between one salt treatment; interaction shows significantly different interactions between salt treatments and light regimes tested by two-way ANOVA, followed by post-hoc Bonferroni test (p ≤ 0.05) (** ≤ 0.01, *** ≤ 0.001); n = 8 pools of three individual plants each from two independent experiments.

ns, not significant.

Considering the plant response to the light regime, *C. officinalis* exhibited a higher content of chlorophyll *a* and *b* at all salt levels in LR1 (greenhouse) compared with LR2 (indoor farming), which was at no salt 0.2-fold higher and at 600 mM of salt 3.1-fold higher. Considering the plant response to salt treatment, we found that the content of chlorophyll *a* and *b* decreased in both light regimes. This decrease occurred in LR2 (indoor farming) from 200 mM of salt but was only observed in LR1 (greenhouse) at higher salinity (600 mM). However, due to the changes in water content, the results based on the fresh mass basis are different. For instance, the highest content of chlorophylls was found at 600 mM of salt in LR1 (greenhouse) and corresponds to the darker green color of the leaves (**Figure S8**).

Considering the plant response to the light regime, *A. hortensis* had a 0.2-fold increased content of chlorophyll *a* at no salt in LR2 (indoor farming), and in LR1 (greenhouse), there was an increased content of both chlorophylls at 200 and 600 mM of salt. Considering the plant response to salt treatment, we determined that the response was similar to *C. officinalis* in LR1 (greenhouse), expressed in a decreased content at 600 mM of salt in both chlorophylls. In LR2 (indoor farming), however, the decrease of both chlorophylls was already observed at 50 mM of salt. The impact was more pronounced on a fresh mass basis. The treatment with 600 mM of salt resulted in a reduction, independent of the light regime. The effect was even more evident under LR1 (greenhouse) and significantly induced chlorophyll reduction starting from 50 mM of salt.

Considering the plant response to the light regime, *S. europaea* in LR2 (indoor farming) showed a drastically higher chlorophyll *a* content in the salt treatments than without salt. Considering the plant response to salt treatment, we found that the lowest content of both chlorophylls could be measured at no salt and then a steep increase at 50 mM, in both light regimes, but differed in the intensity of the increase. Chlorophyll *a* showed at 50 mM of salt in LR2 (indoor farming) a 10-times higher increase than in LR1 (greenhouse). Although these differences in content between LR1 (greenhouse) and LR2 (indoor farming) decreased with increasing salinity, at 600 mM of salt, the difference in contents had decreased by half. At 50, 200, and 600 mM of salt, we also observed a higher chlorophyll *a* content in LR2 (indoor farming) on a fresh mass basis.

For all halophyte species and both chlorophylls, we observed a significant interaction between the light regime and salt treatment on a dry mass basis. On a fresh mass basis, this interaction was observed for *A. hortensis* and *C. officinalis*, but not for *S. europaea*.

#### Content of individual carotenoids in the leaves

The individual carotenoids showed differences in their content related to the response to salt treatment and light regime and related to the plant species (**Table 3**, dry mass basis; **Table S6**, fresh mass basis).

**Table 3:** Content of carotenoids on a dry mass basis in the leaves of 6- to 9-week-old plants (means ± SEM).

		Salt treatment (mM NaCl)	Lutein (ng mg^−1^ DM)					β-Carotene (ng mg^−1^ DM)					Zeaxanthin (ng mg^−1^ DM)					*all-trans*-Violaxanthin (ng mg^−1^ DM)					*9Z*-Neoxanthin (ng mg^−1^ DM)				
*Cochlearia officinalis*	Light regime 1	No salt	1,019.02	±	47.83	a	***	300.39	±	19.36	b		14.39	±	1.02	ns		204.73	±	40.98	a		245.43	±	16.39	ab	**
		50	1,080.23	±	29.39	a	***	396.59	±	6.95	a		19.39	±	2.54	ns		181.14	±	33.45	ab		268.29	±	7.79	a	***
		200	1,004.56	±	38.25	a	***	428.78	±	8.66	a	***	29.55	±	6.34	ns		156.77	±	19.06	ab		225.08	±	9.76	b	***
		600	534.79	±	30.43	b	***	277.09	±	28.21	b	***	29.93	±	3.35	ns		92.81	±	23.10	b		147.74	±	17.67	c	***
	Light regime 2	No salt	821.81	±	17.80	A		360.76	±	10.52	A	*	10.42	±	0.90	b		218.13	±	30.66	A		201.66	±	4.68	A	
		50	759.90	±	10.42	A		397.04	±	6.43	A		17.15	±	1.02	ab		254.93	±	10.16	A	*	178.75	±	4.53	A	
		200	542.08	±	10.94	B		291.83	±	3.73	B		32.31	±	2.33	a		118.21	±	4.02	B		105.69	±	4.44	B	
		600	197.46	±	31.20	C		81.68	±	14.39	C		19.25	±	3.35	a		27.51	±	4.88	C		34.80	±	7.26	C	
Interaction: light regime × salt treatment							***					***					ns					***					**
*Atriplex hortensis*	Light regime 1	No salt	291.13	±	3.90	a		155.39	±	4.33	b		6.57	±	0.82	b		123.76	±	16.61	ns		72.80	±	0.98	a	*
		50	304.76	±	6.71	a	*	186.99	±	4.64	ab		12.96	±	3.57	b		121.23	±	5.63	ns		72.52	±	1.75	a	***
		200	286.67	±	6.46	a	***	218.25	±	2.98	a		30.26	±	6.05	a	**	103.88	±	7.97	ns		71.07	±	0.65	a	***
		600	116.97	±	12.89	b	***	115.41	±	15.48	c	***	16.26	±	0.67	b	*	92.92	±	12.97	ns	**	38.73	±	4.10	b	***
	Light regime 2	No salt	326.36	±	12.42	A	*	262.65	±	10.22	A	***	11.15	±	1.16	ns		134.15	±	45.22	A		55.61	±	7.15	A	
		50	272.81	±	3.36	B		250.73	±	3.69	A	***	8.90	±	2.35	ns		100.86	±	24.99	AB		36.45	±	9.14	B	
		200	211.79	±	10.37	C		194.80	±	10.23	B		26.09	±	8.03	ns		58.80	±	5.96	B		35.88	±	6.86	B	
		600	33.71	±	6.39	D		32.99	±	6.50	C		4.96	±	0.67	ns		14.15	±	0.89	C		11.72	±	1.14	C	
Interaction: light regime × salt treatment							***					***					**					*					ns
*Salicornia europaea*	Light regime 1	No salt	48.68	±	17.69	b		17.95	±	2.26	b		25.54	±	1.68	a	***	21.12	±	4.71	ns		22.81	±	1.78	b	
		50	121.78	±	17.81	ab		32.24	±	9.45	b		6.38	±	1.61	b		21.31	±	3.29	ns		23.81	±	1.81	b	
		200	217.64	±	17.73	a		72.81	±	4.86	a		8.48	±	1.96	b		17.07	±	1.22	ns		46.99	±	3.61	a	
		600	139.13	±	14.68	b		28.29	±	6.58	b		6.19	±	0.60	b		15.95	±	1.02	ns		34.05	±	2.39	b	
	Light regime 2	No salt	55.70	±	11.10	C		8.26	±	1.14	C		0.96	±	0.18	ns		17.88	±	1.13	B		19.39	±	2.40	D	
		50	364.75	±	18.03	A	***	159.50	±	13.59	A	***	5.97	±	0.36	ns		40.86	±	9.52	A	**	77.97	±	4.57	A	***
		200	337.14	±	14.50	A	***	144.03	±	5.53	A	***	6.26	±	0.59	ns		41.75	±	3.98	A	***	65.67	±	2.69	B	***
		600	207.47	±	8.07	B	*	82.39	±	3.43	B	***	4.84	±	0.60	ns		38.76	±	3.60	A	**	38.85	±	3.91	C	
Interaction: light regime × salt treatment							***					***					***					*					***

Small letters indicate significant differences between salt treatments in light regime 1 (LR1, greenhouse) in alphabetical order from highest to lowest; capital letters indicate significant differences between salt treatments in light regime 2 (LR2, indoor farming) in alphabetical order from highest to lowest; asterisks indicate significant differences between LR1 and LR2 in between one salt treatment; interaction shows significantly different interactions between salt treatments and light regimes tested by two-way ANOVA, followed by post-hoc Bonferroni test (p ≤ 0.05) (* ≤ 0.05, ** ≤ 0.01, *** ≤ 0.001); n = 8 pools of three individual plants each from two independent experiments.

ns, not significant.

Lutein displayed a similar response as chlorophylls to salt treatment and light regime for all plant species. Only for *A. hortensis*, we observed, in addition to the higher content at 200 and 600 mM of salt in LR1 (greenhouse) compared with LR2 (indoor farming), also at 50 mM of salt a higher content in LR1 (greenhouse). Likewise, changes on a fresh mass basis were observed, and for all halophyte species, the highest levels were found for 200 or 600 mM of salt, except for *C. officinalis*, where no significant changes were found under LR2 (indoor farming).

β-Carotene showed the same pattern in both *C. officinalis* and *A. hortensis* but with a different intensity. Considering the plant response to the light regime, we found that at no salt both halophytes showed higher content in LR2 (indoor farming), and the content was 0.7-fold higher in *A. hortensis* and 0.2-fold higher in *C. officinalis*. Considering the plant response to salt treatment, we found that both plant species showed in LR1 (greenhouse) an increasing content from no salt to 200 mM of salt and then a decrease again at 600 mM to the no-salt treatment, whereas, in LR2 (indoor farming), the content was the highest in the no salt and 50 mM and then decreased. Considering the plant response to the light regime, *S. europaea* exhibited a higher content of β-carotene at 50, 200, and 600 mM of salt in LR2 (indoor farming) than in LR1 (greenhouse). Considering the plant response to salt treatment, we found that *S. europaea* showed in LR1 (greenhouse) an increase at 200 mM and in LR2 (indoor farming) a steep increase from no salt to 50 mM and then a decrease at 600 mM again, although at 600 mM, the content was still higher compared with the no salt. Based on fresh mass, the highest β-carotene content under LR1 (greenhouse) was found for *C. officinalis* at 600 mM and for *A. hortensis* at 200 mM of salt, whereas no significant changes were detected for S*. europaea*. In LR2 (indoor farming), 50 mM of salt induced the highest accumulation rate in *C. officinalis*, 50 and 200 mM in *A. hortensis*, and 50 to 600 mM in *S. europaea.* These changes are also reflected in the significant interactions of light regime and salt observed for β-carotene and for all the halophytes (**Table S6**).

For zeaxanthin, we observed a very indifferent pattern. Considering the plant response to salt treatment, we found that *C. officinalis* only in LR2 (indoor farming) expressed significantly increased content at 200 and 600 mM of salt. Furthermore, *A. hortensis* showed only in LR1 (greenhouse) an increased content at 200 mM of salt. Considering the plant response to the light regime, *A. hortensis* displayed a higher content in LR1 (greenhouse) at 200 and 600 mM of salt. Considering the plant response to the light regime, *S. europaea* exhibited a higher content at no salt in LR1 (greenhouse), and considering the plant response to salt treatment, it showed a decreased content at 50, 200, and 600 mM of salt in LR1 (greenhouse). On a fresh mass basis, the only difference was an increased content at 600 mM of salt in LR1 (greenhouse) for *C. officinalis.*

For both *C. officinalis* and *A. hortensis*, violaxanthin showed a decreasing trend with increasing salinity in both light regimes. Considering the plant response to the light regime, *A. hortensis* showed only at 600 mM a higher content in LR1 (greenhouse) and *C. officinalis* at 50 mM in LR2 (indoor farming). Furthermore, *S. europaea* exhibited an increased content in LR2 (indoor farming) within salinity levels. Considering the plant response to salt treatment, we observed only in LR2 (indoor farming) a significant response, expressed with an increased content from 50 to 600 mM of salt. On a fresh mass basis, *A. hortensis* showed no significant differences, while *C. officinalis* showed a contrasting pattern in LR1 (greenhouse) and the same pattern in LR2 (indoor farming). Also, comparing the light regimes, we observed a higher content at no salt in LR2 (indoor farming) and at 600 mM in LR1 (greenhouse). For *S. europaea*, we observed an increased content at 200 mM in LR2 (indoor farming) compared with all salt treatments.

For both facultative halophytes (*C. officinalis* and *A. hortensis*), neoxanthin presented a strong response to salt treatment and light regime. Considering the plant response to the light regime, the content for both plants was higher at all salt levels in LR1 (greenhouse). Considering the plant response to salt treatment, within increasing salinity, the content decreased, whereby the decrease in LR2 (indoor farming) was much steeper. For the obligate halophyte *S. europaea*, considering its response to the light regime, we observed a higher content in LR2 (indoor farming) at 50 and 200 mM of salt. Based on fresh mass, *A. hortensis* again showed no significant differences, *S. europaea* the same pattern, and *C. officinalis* in LR1 (greenhouse) the highest content at 600 mM, and in LR2 (indoor farming), there were no significant differences.

Taken together, we observed a similar pattern for both facultative halophytes, *C. officinalis* and *A. hortensis*, which was different from *S. europaea*. Also, lutein and neoxanthin showed the same response to salt treatment and light regime, which differed from the response in β-carotene, whereas zeaxanthin and violaxanthin showed the most indifferent pattern. Both lutein and β-carotene showed an interaction between the light regime and salt treatment for all plant species, zeaxanthin only for *A. hortensis* and *S. europaea*, and neoxanthin only for *C. officinalis* and *S. europaea* on a dry mass basis. In contrast, on a fresh mass basis, no interaction was observed for lutein for *A. hortensis* and *S. europaea*.

### Impact on the overall metabolite composition

**Figure 4:**
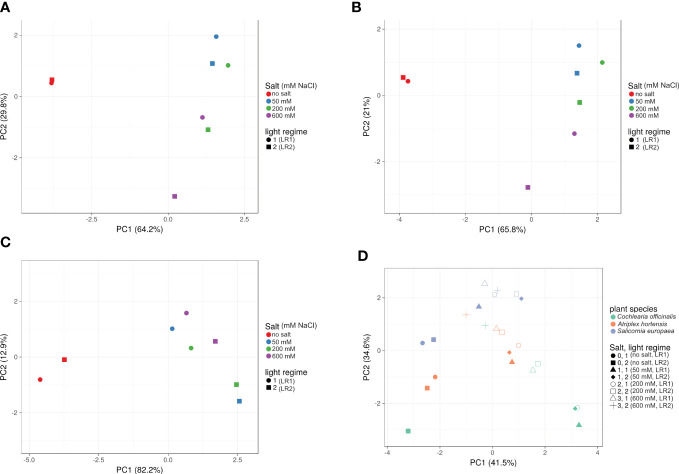
Effect of salt treatment and light regime on metabolite profile. PCA plots of **(A)** *Cochlearia officinalis*, **(B)** *Atriplex hortensis*, **(C)** *Salicornia europaea*, and **(D)** all three plant species. PCA plots were generated with ClustVis; *n* = 8 pools of three individual plants each from two independent experiments; unit variance was applied to rows; SVD with imputations is used to calculate principal components; factor loadings are provided in *Appendix* **(B)** Light regime 1 (LR1, greenhouse); light regime 2 (LR2, indoor farming).

To gain insight into the influence of light regime and salt treatment on the dynamic metabolic variation, a PCA analysis was performed (**Figure 4**). The greatest influence was due to the difference in salt treatment. For all three halophytes (*C. officinalis*, *A. hortensis*, and *S. europaea*), distinct clusters were found for the treatments with and without salt based on PC1 and PC2 (**Figures 4A–C**). With respect to the light regime, the effects on the metabolite profiles were less pronounced. However, interactions on individual metabolite levels have been demonstrated, e.g., for carotenoids and chlorophylls (**Tables 2**, **3**).

## Discussion

Here, we demonstrated that the interaction of light regime and salt treatment modulates the content of photosynthetic pigments and influences the salt tolerance of halophytes.

### Influence of light regime on the response to salt treatment

In the evaluation of salt-tolerant crops, the yield loss in response to the salt concentrations in soil or water is a key aspect to be considered. Considering the salt treatments, a reduction in fresh mass was found at 50/200 mM for *C. officinalis*, at 200 mM for *A. hortensis*, and at 600 mM and no salt for *S. europaea*. Evaluating the effect of the light regime in the non-salt-stressed conditions (*A. hortensis* and *C. officinalis* no salt and for *S. europaea* 50 and 200 mM of salt), the fresh mass is higher in indoor farming (LR2). This suggests that the DLI in the greenhouse (LR1) was too low for optimal growth. However, relative fresh mass still differs in the light regimes at salt treatments 50 and 200 mM for *S. europaea* and *C. officinalis* (**Figure S7**). Additionally, we observed differences in the influence of salt treatment on the water content, between both the facultative halophytes (*A. hortensis* and *C. officinalis*) and the obligate halophyte (*S. europaea*). Water content was less affected by salt treatment for the obligate halophyte. Succulent halophytes (salt-accumulating), like *Suaeda maritima*, show a different osmotic adjustment and, thus, a different water content under salinity (Flowers and Colmer, 2008). To evaluate the influence of light regime in relation to salt treatment, further insights were obtained by studying changes in the ABA and chloride contents. ABA serves as an indicator of salt stress response in halophytes and glycophytes and mediates the stomatal movement of guard cells (Zhang et al., 2006; Karimi et al., 2021). According to previous research, we observed a correlation between increased salt stress (increased ABA content) and yield loss (Breckle, 2002; Metselaar, 2013). However, the response of ABA differs between halophytes and glycophytes, at least with respect to the salt level. A study conducted by Karimi et al. (2021) showed only a short-term response of ABA in *Thellungiella salsuginea* (a halophyte) at 200 mM, while *Arabidopsis thaliana* (a glycophyte) showed a long-term response. Ben Hassine et al. (2009), on the other hand, showed an increased ABA content in the seedlings of the halophyte *Atriplex halimus* in a short- and long-term response to 160 mM of NaCl treatment. This suggests that ABA regulation not only differs among glycophytes and halophytes but also among halophyte species. Aside from sodium content, chloride content also changes with salinity treatment, and accumulation varies between halophyte species. For instance, the ratio of sodium and potassium cations to chloride anions varies among halophyte species, which could influence the external chloride uptake (Flowers and Colmer, 2008) and should be considered in future studies.

In this study, *C. officinalis* showed an increased ABA content correlating with increased chloride and reduced growth already from 50/200 mM. This is in accordance with a lower salinity tolerance (de Vos and Broekman, 2013). In contrast, de Vos and Broekman (2013) observed a higher percent dry weight at 200 mM and a leaf succulence, whereas, in our study, it was only significantly increased at 600 mM. The lowest chloride accumulation in the leaves of *C. officinalis* compared with the two other plant species (*A. hortensis* and *S. europaea*) would support the salt exclusion mechanism if the salt is translocated into the xylem and root (Chen et al., 2018). *Atriplex hortensis* showed an increased ABA content (only in LR1), paired with a reduction in fresh mass and chloride accumulation at 200 mM of salt. This is in accordance with the literature, where a salt tolerance of up to 250 mM of salt was shown for another variety of *A. hortensis* (red orache) (Wilson et al., 2000). Furthermore, we observed excreted salt crystals on the leaf surface, which is typical for halophytes with salt bladders (Schirmer and Breckle, 1982). Therefore, when considering chloride content in *A. hortensis*, it is important to consider the salt deposition on the leaf surface. One possibility is to wash off the salt from the leaves before measurement, but this may not reveal the transport of salt into the leaves, making it more difficult to compare salt tolerance mechanisms. Nevertheless, it would be interesting to distinguish between the salt excreted and the salt accumulated from and in the leaf. Since *S. europaea* is an obligate halophyte, it showed an increase in ABA and a decrease in fresh mass not only at 600 mM but also at no salt, unlike the other plant species, suggesting that this salt concentration and very low salt lead to stress. This can be explained by the fact that in obligate halophytes, salt uptake is essential for maintaining turgor and for optimal growth and is also reflected in the water content, which changes only slightly with salt treatment (Glenn and O’Leary, 1984). Furthermore, when considering the differences between chloride content in shoots based on dry and fresh mass, it can be clearly observed that *S. europaea* has a lower chloride/fresh mass ratio at higher salt treatments compared with the other plant species. This is due to lower salt-induced water loss, indicating better osmotic regulation in *S. europaea* at higher salinity levels.

Whereas the influence of the light regime on fresh mass was clearly visible, the effect on metabolite profiles was less pronounced. However, the influence of the light regime on metabolite profiles also differed within plant species (**Figure 4**). Again, different patterns were observed with respect to salinity treatment, suggesting a different adaptation to salinity and a different influence of the interaction between salinity treatment and light regime in different halophyte species. This interaction is particularly interesting for photosynthetic pigments.

### The interaction of light regime and salt treatment in influencing photosynthetic pigments

Carotenoids and chlorophylls have multiple functions in plants; for example, carotenoids are accessory pigments, and also they have essential photoprotective properties, while chlorophylls are the main pigments of photosystems. Carotenoid and chlorophyll biosynthesis and metabolism are affected by light, e.g., light quality or light intensity, as well as salinity (Pizarro and Stange, 2009; Tanaka and Tanaka, 2011; Soltabayeva et al., 2021). All the pigments studied are part of the photochemical apparatus but have different functions according to which they can be divided into two groups. First, chlorophyll *a* and *b*, lutein, and neoxanthin, in a simplified way, function as absorbers and converters for the incoming light energy (Choudhury and Behera, 2001). Second, violaxanthin, β-carotene, and zeaxanthin, on the other hand, function as dissipators of excessive light energy (Choudhury and Behera, 2001). This should be taken into account as we observed a different pattern in pigment accumulation between the two light regimes in the salt stress and salt tolerance range.

Salt stress leads to limited activity in several parts of the photosynthetic apparatus (e.g., RuBisCO activity, NADPH oxidase activity) and, thus, increased formation of reactive oxygen species (ROS) (Hasanuzzaman et al., 2020). In high light stress, the high photon flux density leads to excessive light energy that exceeds the capacity of the photosynthetic apparatus, resulting in the formation of ROS, which can cause subcellular damage and photooxidation of pigments (Gilmore, 2001). A study by Simkin et al. (2003) showed that in pepper (*Capsicum annuum* L. cv. Yolo Wonder), the photooxidation of carotenoids already occurs at the transition of light intensity from 150 to 280 µmol m^−2^ s^−1^. Since we observed a combined effect of light regime and salt treatment and found a strong difference in DLI between the two light regimes, this difference must be taken into account. Higher light energy combined with the salt stress-induced limited activity of the photosynthetic apparatus results in overexcitation of the photosynthetic apparatus and increased ROS formation that exceeds antioxidant capacity (Carillo, 2018). Accordingly, we observed a salt stress-induced reduction of all pigments in indoor farming (LR2) with a higher DLI. In contrast, within salt tolerance ranges, we observed an accumulation of the carotenoids violaxanthin, β-carotene, and zeaxanthin, which act as dissipators of excess light energy and thus scavenge ROS, and a decrease in lutein and neoxanthin and chlorophylls, which act as absorbers and converters of incoming light energy and thus maintain photosynthetic activity (Choudhury and Behera, 2001). These effects are in accordance with the changes in our study in indoor farming (LR2) and resulted in higher levels of violaxanthin, β-carotene, and zeaxanthin during salt stress at lower DLI in greenhouse (LR1) and suggest that DLI affects the carotenoid profiles as a function of salt concentration with respect to their different functions in the photosynthetic process.

Interestingly, the response of the obligate halophyte (*S. europaea*) in salt stress (no salt) is different from the response of the facultative halophytes (*C. officinalis* and *A. hortensis*). For *S. europaea*, we observed particularly low contents of pigments in both light regimes at no salt. An explanation could be a different adaptation of the photosynthetic apparatus to salt. It is suggested that halophytes have the ability to regulate steady chloride concentrations by a different ion (Na^+^, Cl^−^, and K^+^) transport compared with glycophytes (Bose et al., 2017). Since salt is essential for maintaining intracellular pH, altered pH in the thylakoid interior could affect the function of crucial enzymes for photosynthesis, e.g., RuBisCO or NADPH oxidase. This could influence photosynthetic activity, e.g., photooxidation of pigments and biosynthesis of carotenoids and chlorophylls (Glenn and O’Leary, 1984).

Taken together, we observed an interaction of light regime and salt treatment in influencing the performance of the three halophyte species. Therefore, when optimizing the light conditions in indoor farming, the plant species, salt tolerance, and salinity of the cultivation medium must be taken into account. In indoor farming, lighting conditions are not only important for the plants but also for evaluating the profitability and sustainability of a production system. Hence, light efficiency use (LUE) is a factor, considering the consumed electricity of the system, which helps to compare indoor farming and greenhouse cultivation. A study by Jin et al. (2022) pointed out that the average LUE in vertical farming is higher than in greenhouse cultivation. Considering the influence of salt stress and light on yield, assuming LUE is higher under lower DLI (greenhouse) than under higher DLI (indoor farming), therefore, lower light intensity in saline indoor farming could decrease light energy while maintaining yield and, thus, optimize LUE. Nevertheless, the DLI in the greenhouse (LR1) was also too low. Therefore, lower DLI with moderate salinity could lead to optimized resource use and even improved nutritional quality by increasing the amount of PSMs. The implementation of UV-B LEDs or colored LEDs could further enhance the PSM content (Wiesner-Reinhold et al., 2021; Frede and Baldermann, 2022). Further research could aim to study the influence of DLI and salinity on other nutritive compounds, such as polyphenols and vitamins.

### Study limitations and perspective

The major limitation of this study is that the effects are assumed to be due to the daily light integral and not due to light quality. An altered light quality, in this case mainly light spectra, has also an influence on the plant metabolism and pigment content (Alrifai et al., 2019; Frede et al., 2019). For example, different photoreceptors can be activated through changes in the light spectra (Kami et al., 2010). However, our study design aimed to investigate the differences between greenhouse cultivation and indoor farming, and thus, there are differences not only in the light regime but also in light intensity. Since the daily light impact was highly influenced (72% differences between both light regimes) by the light regimes, we focused on this while explaining the results. Nevertheless, it would be beneficial to investigate further influences of different light parameters on the quality of vegetables in indoor farming systems.

Regarding the response of plants to salt stress, it is important to know whether salt was applied in a single step or gradually. If salt is applied in a single step, there is a possibility that plants will suffer from salt shock (Shavrukov, 2012). In our study, salt was applied in a single step. However, salt was applied in an NFT system where the pots were irrigated from below, which resulted in a slower accumulation of salt in the soil. In addition, plants had a long acclimation period of 17 days, during which they could have recovered from the osmotic shock (Shavrukov, 2012). Nevertheless, this is an important point that should be considered in future studies which may affect the tolerance of plants to ionic stress and, hence, their response to varying light regimes.

It would be interesting to study the modification of light conditions with respect to the adjustable salt tolerance of halophytes and the impact of light in relation to the use of different saline water sources. The salt concentration is not only dependent on the water source, e.g., brackish water, wastewater, or brine water, but also on the location (Atkinson and Bingman, 1997). For example, regional brine waters have different salt concentrations and compositions (Fitzner et al., 2021). One option to adjust the salt concentration to the halophyte salt tolerance range is dilution with freshwater. However, freshwater is an exhaustible resource, and in sustainable agriculture, freshwater consumption should be reduced (Gleick, 1993). If there is a way to regulate light intensity, this would be a potential solution.

Further research also could aim to study other halophyte species to broaden the picture of differences between obligate and facultative halophytes and investigate the interaction of the salt tolerance mechanism and the influence of light.

In conclusion, this study highlights the potential applications of halophytes for indoor farming and also hints at the adaptation of photosynthesis during salt stress under different light regimes in halophytes. Furthermore, optimization of indoor farming lighting conditions, taking into account salinity and plant species, could improve resource efficiency and pigment profile. Given the limited land and water use and the changing climate conditions, we argue that indoor farming has a high potential to become a fundamental contributor to global agriculture. In addition to sustainable crop production, healthy and sustainable nutrition will be a valued aspect of future diets. Halophytes are not only suitable for indoor farming but can also be irrigated with saline water, which conserves freshwater resources, and are additionally rich in PSM. Hence, saline indoor farming with halophytes could contribute to food and nutritional security in the future.

## Data availability statement

The raw data supporting the conclusions of this article will be made available by the authors, without undue reservation.

## Author contributions

MF performed the experiment, analysis, and data analysis. MF, MS, and SB wrote the article. MF and SB developed and established the methods. MS and SB did the funding acquisition. MS and SB conceived the original research plans. All authors contributed to the article and approved the submitted version.

## References

[B1] Abdelly C. Flowers T. Gul B. Koyro H.-W. Savouré A. (2022). Recent advances in research on halophytes: From fundamental to applied aspects. Environ. Exp. Bot. 193, 104683. doi: 10.1016/j.envexpbot.2021.104683

[B2] Aghaleh M. (2011). Effect of salt stress on physiological and antioxidative responses in two species of *Salicornia* (*S. persica* and *S. europaea*). Acta Physiol. Plant 33, 1261–1270. doi: 10.1007/s11738-010-0656-x

[B3] Alrifai O. Hao X. Marcone M. F. Tsao R. (2019). Current review of the modulatory effects of LED lights on photosynthesis of secondary metabolites and future perspectives of microgreen vegetables. J. Agric. Food Chem. 67, 6075–6090. doi: 10.1021/acs.jafc.9b00819 31021630

[B4] Annunziata M. G. Apelt F. Carillo P. Krause U. Feil R. MENGIN V.. (2017). Getting back to nature: A reality check for experiments in controlled environments. J. Exp. Bot. 68, 4463–4477. doi: 10.1093/jxb/erx220 28673035 PMC5853417

[B5] Araus J. L. Rezzouk F. Z. Thushar S. Shahid M. Elouafi I. A. Bort J.. (2021). Effect of irrigation salinity and ecotype on the growth, physiological indicators and seed yield and quality of *Salicornia europaea* . Plant Sci. 304, 110819. doi: 10.1016/j.plantsci.2021.110819 33568309

[B6] Asseng S. Guarin J. R. Raman M. Monje O. Kiss G. Despommier D. D.. (2020). Wheat yield potential in controlled-environment vertical farms. Proc. Natl. Acad. Sci. U.S.A. 117, 19131–19135. doi: 10.1073/pnas.2002655117 32719119 PMC7430987

[B7] Atkinson M. Bingman C. (1997). Elemental composition of commercial seasalts. Journal of Aquariculture and Aquatic Sciences. 8, 39–43.

[B8] Ben Hassine A. Ghanem M. E. Bouzid S. Lutts S. (2009). Abscisic acid has contrasting effects on salt excretion and polyamine concentrations of an inland and a coastal population of the Mediterranean xero-halophyte species *Atriplex halimus* . Ann. Bot. 104, 925–936. doi: 10.1093/aob/mcp174 19666900 PMC2749539

[B9] Björn L. O. Papageorgiou G. C. Blankenship R. E. Govindjee (2009). A viewpoint: Why chlorophyll a? Photosynth. Res. 99, 85–98. doi: 10.1007/s11120-008-9395-x 19125349

[B10] Bose J. Munns R. Shabala S. Gilliham M. Pogson B. Tyerman S. D. (2017). Chloroplast function and ion regulation in plants growing on saline soils: Lessons from halophytes. J. Exp. Bot. 68, 3129–3143. doi: 10.1093/jxb/erx142 28472512

[B11] Breckle S. W. (2002). Salinity, halophytes and salt affected natural ecosystems (Springer Dordrecht).

[B12] Carillo P. (2018). GABA shunt in durum wheat. Front. Plant Sci. 9, 100. doi: 10.3389/fpls.2018.00100 29456548 PMC5801424

[B13] Chen M. Yang Z. Liu J. Zhu T. Wei X. Fan H.. (2018). Adaptation mechanism of salt excluders under saline conditions and its applications. Int. J. Mol. Sci. 19. doi: 10.3390/ijms19113668 PMC6274768 30463331

[B14] Choudhury N. K. Behera R. K. (2001). Photoinhibition of photosynthesis: Role of carotenoids in photoprotection of chloroplast constituents. Photosynthetica 39, 481–488. doi: 10.1023/A:1015647708360

[B15] de Vos A. C. Broekman R. (2013). Developing and testing new halophyte crops: A case study of salt tolerance of two species of the brassicaceae, *Diplotaxis tenuifolia* and cochlearia officinalis. Environ. Exp. Bot. 92, 154–164. doi: 10.1016/j.envexpbot.2012.08.003

[B16] Ellouzi H. Hamed K. B. Hernández I. Cela J. Müller M. Magné C.. (2014). A comparative study of the early osmotic, ionic, redox and hormonal signaling response in leaves and roots of two halophytes and a glycophyte to salinity. Planta 240, 1299–1317. doi: 10.1007/s00425-014-2154-7 25156490

[B17] Errard A. Ulrichs C. Kühne S. Mewis I. Drungowski M. Schreiner M.. (2015). Single- versus multiple-pest infestation affects differently the biochemistry of tomato (*Solanum lycopersicum* ‘Ailsa craig’). J. Agric. Food Chem. 63, 10103–10111. doi: 10.1021/acs.jafc.5b03884 26507319

[B18] FAO (2020). “The state of food and agriculture 2020,” in Overcoming water challanges in agriculture (Rome: FAO).

[B19] Fiedor J. Burda K. (2014). Potential role of carotenoids as antioxidants in human health and disease. Nutrients 6, 466–488. doi: 10.3390/nu6020466 24473231 PMC3942711

[B20] Fitzner M. Fricke A. Schreiner M. Baldermann S. (2021). Utilization of regional natural brines for the indoor cultivation of *Salicornia europaea* . Sustainability 13, 12105. doi: 10.3390/su132112105

[B21] Flowers T. J. Colmer T. D. (2008). Salinity tolerance in halophytes. New Phytol. 179, 945–963. doi: 10.1111/j.1469-8137.2008.02531.x 18565144

[B22] Frede K. Baldermann S. (2022). Accumulation of carotenoids in brassica rapa ssp. *chinensis* by a high proportion of blue in the light spectrum. Photochem. Photobiol. Sci. doi: 10.1007/s43630-022-00270-8 35895283

[B23] Frede K. Ebert F. Kipp A. P. Schwerdtle T. Baldermann S. (2017). Lutein activates the transcription factor Nrf2 in human retinal pigment epithelial cells. J. Agric. Food Chem. 65, 5944–5952. doi: 10.1021/acs.jafc.7b01929 28665123

[B24] Frede K. Schreiner M. Baldermann S. (2019). Light quality-induced changes of carotenoid composition in pak choi brassica rapa ssp. chinensis. J. Photochem. Photobiol. B 193, 18–30. doi: 10.1016/j.jphotobiol.2019.02.001 30798151

[B25] Frede K. Schreiner M. Zrenner R. Graefe J. Baldermann S. (2018). Carotenoid biosynthesis of pak choi (*Brassica rapa* ssp. *chinensis*) sprouts grown under different light-emitting diodes during the diurnal course. Photochem. Photobiol. Sci. 17, 1289–1300. doi: 10.1039/c8pp00136g 30065986

[B26] Gilmore A. M. (2001). Xanthophyll cycle-dependent nonphotochemical quenching in photosystem II: Mechanistic insights gained from *Arabidopsis thaliana* l. mutants that lack violaxanthin deepoxidase activity and/or lutein. Photosyn. Res. 67, 89–101. doi: 10.1023/A:1010657000548 16228319

[B27] Gleick P. H. (1993). Water in crisis: A guide to the world's fresh water resources (Oxford Univ Pr: New York).

[B28] Glenn E. P. O’Leary J. W. (1984). Relationship between salt accumulation and water content of dicotyledonous halophytes. Plant Cell Environ. 7, 253–261. doi: 10.1111/1365-3040.ep11589448

[B29] Golldack D. Li C. Mohan H. Probst N. (2014). Tolerance to drought and salt stress in plants: Unraveling the signaling networks. Front. Plant Sci. 5, 151. doi: 10.3389/fpls.2014.00151 24795738 PMC4001066

[B30] Hasanuzzaman M. Bhuyan M. Parvin K. Bhuiyan T. F. Anee T. I. Nahar K.. (2020). Regulation of ROS metabolism in plants under environmental stress: A review of recent experimental evidence. Int. J. Mol. Sci. 21. doi: 10.3390/ijms21228695 PMC7698618 33218014

[B31] Hasanuzzaman M. Nahar K. Alam M. M. Bhowmik P. C. Hossain M. A. Rahman M. M.. (2014). Potential use of halophytes to remediate saline soils. Biomed. Res. Int. 2014, 589341. doi: 10.1155/2014/589341 25110683 PMC4109415

[B32] He Q. Silliman B. R. Cui B. (2017). Incorporating thresholds into understanding salinity tolerance: A study using salt-tolerant plants in salt marshes. Ecol. Evol. 7, 6326–6333. doi: 10.1002/ece3.3209 28861236 PMC5574752

[B33] Heinze M. Hanschen F. S. Wiesner-Reinhold M. Baldermann S. Grafe J. Schreiner M.. (2018). Effects of developmental stages and reduced UVB and low UV conditions on plant secondary metabolite profiles in pak choi (*Brassica rapa* subsp. *chinensis*). J. Agric. Food Chem. 66, 1678–1692. doi: 10.1021/acs.jafc.7b03996 29397716

[B34] Hernandez Velasco M. (2021). Enabling year-round cultivation in the nordics-agrivoltaics and adaptive LED lighting control of daily light integral. Agriculture 11, 1255. doi: 10.3390/agriculture11121255

[B35] Hideg E. Barta C. Kálai T. Vass I. Hideg K. Asada K. (2002). Detection of singlet oxygen and superoxide with fluorescent sensors in leaves under stress by photoinhibition or UV radiation. Plant Cell Physiol. 43, 1154–1164. doi: 10.1093/pcp/pcf145 12407195

[B36] Jin W. Lopez F. ,. D. Heuvelink E. Marcelis L. F. M. (2022). Light use efficiency of lettuce cultivation in vertical farms compared with greenhouse and field. Food Energy Secur. 12, e391. doi: 10.1002/fes3.391

[B37] Julkowska M. M. Testerink C. (2015). Tuning plant signaling and growth to survive salt. Trends Plant Sci. 20, 586–594. doi: 10.1016/j.tplants.2015.06.008 26205171

[B38] Kachout S. S. Mansoura B. A. Jaffel K. Leclerc J. C. Rejeb M. N. Ouerghi Z. (2009). The effect of salinity on the growth of the halophyte *Atriplex hortensis* (*Chenopodiaceae*). Appl. Ecol. Environ. Res. 7, 319–332. doi: 10.15666/aeer/0704_319332

[B39] Kami C. Lorrain S. Hornitschek P. Fankhauser C. (2010). Light-regulated plant growth and development. Curr. Top. Dev. Biol. 91, 29–66. doi: 10.1016/S0070-2153(10)91002-8 20705178

[B40] Karimi S. M. Freund M. Wager B. M. Knoblauch M. Fromm J. ,. M. Mueller H.. (2021). Under salt stress guard cells rewire ion transport and abscisic acid signaling. New Phytol. 231, 1040–1055. doi: 10.1111/nph.17376 33774818

[B41] Kim H.-J. Fonseca J. M. Choi J.-H. Kubota C. KWON D. Y. (2008). Salt in irrigation water affects the nutritional and visual properties of romaine lettuce (*Lactuca sativa* l.). J. Agric. Food Chem. 56, 3772–3776. doi: 10.1021/jf0733719 18439016

[B42] Kim S. Lee E.-Y. Hillman P. F. Ko J. Yang I. Nam S.-J. (2021). Chemical structure and biological activities of secondary metabolites from *Salicornia europaea* l. Molecules 26: 2252. doi: 10.3390/molecules26082252 33924656 PMC8069253

[B43] Ko D. Helariutta Y. (2017). Shoot–root communication in flowering plants. Curr. Biol. 27, R973–R978. doi: 10.1016/j.cub.2017.06.054 28898670

[B44] Korczynski P. C. Logan J. Faust J. E. (2002). Mapping monthly distribution of daily light integrals across the contiguous united states. Horttechnology 12, 12–16. doi: 10.21273/HORTTECH.12.1.12

[B45] Ladeiro B. (2012). Saline agriculture in the 21st century: Using salt contaminated resources to cope food requirements. J. Bot. 2012. doi: 10.1155/2012/310705

[B46] Lado J. Cronje P. Alquézar B. Page A. Manzi M. Gómez-Cadenas A.. (2015). Fruit shading enhances peel color, carotenes accumulation and chromoplast differentiation in red grapefruit. Physiol. Plant 154, 469–484. doi: 10.1111/ppl.12332 25676857

[B47] Lv S. Jiang P. Chen X. Fan P. Wang X. Li Y. (2012). Multiple compartmentalization of sodium conferred salt tolerance in *Salicornia europaea* . Plant Physiol. Biochem. 51, 47–52. doi: 10.1016/j.plaphy.2011.10.015 22153239

[B48] Maina S. Ryu D. H. Cho J. Y. Jung D. S. PARK J. E. Nho C. W.. (2021). Exposure to salinity and light spectra regulates glucosinolates, phenolics, and antioxidant capacity of *Brassica carinata* l. microgreens. Antioxidants 10. doi: 10.3390/antiox10081183 PMC8389028 34439431

[B49] Metselaar E. V. K. (2013). Modelling of soil salinity and halophyte crop production. Environ. Exp. Bot. 92, 186–196. doi: 10.1016/j.envexpbot.2012.10.004

[B50] Naznin M. T. Lefsrud M. Gravel V. Azad M. O. K. (2019). Blue light added with red LEDs enhance growth characteristics, pigments content, and antioxidant capacity in lettuce, spinach, kale, basil, and sweet pepper in a controlled environment. Plants 8. doi: 10.3390/plants8040093 PMC6524371 30965584

[B51] Norton S. C. (2021). Commercial feasibility of indoor saltwater agriculture using saliconia europaea (Charleston, USA: College of Charleston).

[B52] Pizarro L. Stange C. (2009). Light-dependent regulation of carotenoid biosynthesis in plants. Cienc. Investig. Agrar. 36, 143–162. doi: 10.4067/S0718-16202009000200001

[B53] Schirmer U. Breckle S.-W. (1982). “The role of bladders for salt removal in some chenopodiaceae (mainly atriplex species),” in Contributions to the ecology of halophytes. Eds. Sen D. N. Rajpurohit K. S. (Dordrecht: Springer Netherlands).

[B54] Shavrukov Y. (2012). Salt stress or salt shock: Which genes are we studying? J. Exp. Bot. 64, 119–127. doi: 10.1093/jxb/ers316 23186621

[B55] Simkin A. J. Zhu C. Kuntz M. Sandmann G. (2003). Light-dark regulation of carotenoid biosynthesis in pepper (*Capsicum annuum*) leaves. J. Plant Physiol. 160, 439–443. doi: 10.1078/0176-1617-00871 12806770

[B56] Soltabayeva A. Ongaltay A. Omondi J. O. Srivastava S. (2021). Morphological, physiological and molecular markers for salt-stressed plants. Plants 10, 243. doi: 10.3390/plants10020243 33513682 PMC7912532

[B57] Song J. Wang B. (2014). Using euhalophytes to understand salt tolerance and to develop saline agriculture: *Suaeda salsa* as a promising model. Ann. Bot. 115, 541–553. doi: 10.1093/aob/mcu194 25288631 PMC4332605

[B58] Swain M. (2022). “Vertical farming trends and challenges: A new age of agriculture using IoT and machine learning,” in Internet Of things for agriculture 4.0: Impact and challenges (CRC Press).

[B59] Tanaka R. Tanaka A. (2011). Chlorophyll cycle regulates the construction and destruction of the light-harvesting complexes. Biochim. Biophys. Acta Bioenerg. 1807, 968–976. doi: 10.1016/j.bbabio.2011.01.002 21216224

[B60] Trebst A. (2003). Function of β-carotene and tocopherol in photosystem II. Z. für Naturforschung C 58, 609–620. doi: 10.1515/znc-2003-9-1001 14577617

[B61] Tuteja N. (2007). Abscisic acid and abiotic stress signaling. Plant Signal. Behav. 2, 135–138. doi: 10.4161/psb.2.3.4156 19516981 PMC2634038

[B62] van Delden S. H. Sharathkumar M. Butturini M. Graamans L. J. A. Heuvelink E. Kacira M.. (2021). Current status and future challenges in implementing and upscaling vertical farming systems. Nat. Food 2, 944–956. doi: 10.1038/s43016-021-00402-w 37118238

[B63] Ventura Y. Sagi M. (2013). Halophyte crop cultivation: The case for *Salicornia* and *Sarcocornia* . Environ. Exp. Bot. 92, 144–153. doi: 10.1016/j.envexpbot.2012.07.010

[B64] Wiesner-Reinhold M. Dutra G. J. V. Herz C. Tran H. T. T. Baldermann S. Neugart S.. (2021). Subsequent treatment of leafy vegetables with low doses of UVB-radiation does not provoke cytotoxicity, genotoxicity, or oxidative stress in a human liver cell model. Food Biosci. 43, 101327. doi: 10.1016/j.fbio.2021.101327

[B65] Wilson C. Lesch S. M. Grieve C. M. (2000). Growth stage modulates salinity tolerance of new zealand spinach (*Tetragonia tetragonioides*, pall.) and red orach (*Atriplex hortensis* l.). Ann. Bot. 85, 501–509. doi: 10.1006/anbo.1999.1086

[B66] Yuan F. Leng B. Wang B. (2016). Progress in studying salt secretion from the salt glands in recretohalophytes: How do plants secrete salt? Front. Plant Sci. 7. doi: 10.3389/fpls.2016.00977 PMC4927796 27446195

[B67] Zhang J. Jia W. Yang J. Ismail A. M. (2006). Role of ABA in integrating plant responses to drought and salt stresses. Field Crops Res. 97, 111–119. doi: 10.1016/j.fcr.2005.08.018

[B68] Zhu J.-K. (2002). Salt and drought stress signal transduction in plants. Annu. Rev. Plant Biol. 53, 247–273. doi: 10.1146/annurev.arplant.53.091401.143329 12221975 PMC3128348

