# Supplementary material for: The interaction of salinity and light regime modulates photosynthetic pigment content in edible halophytes in greenhouse and indoor farming
Source: Front Plant Sci. 2023 Apr 4;14:1105162. doi: 10.3389/fpls.2023.1105162 (PMC10110887; doi:10.3389/fpls.2023.1105162)
Supplement: Supplementary file 1 [file DataSheet_1.docx]

Supplementary Material

**Supplemental Methods**

**S1.1** Determination of anion content in soil and nutrient solution

Chloride and nitrate content in soil and nutrient solution samples were determined by ion exchange chromatography. Freeze-dried soil samples were grinded (3 times for 2 min, 800 U min^-1^) in a ceramic vessel using a Planetary Ball Mill (PULVERISETTE 7 classic line; Fritsch, Idar-Oberstein, Germany) to a homogenous powder. For extraction, 200 mg fine powder was dissolved in 14 mL ultrapure water and 500 µl of sodium bromide (0.6 mg mL^-1^) was added as an internal standard. The sample preparation of nutrient solution samples was done according to Fitzner et al. (2021). Further extraction and measurement was carried out according to “Determination of chloride concentration in leaves” in section 2.4 of the main manuscript.

**Supplemental Tables**

**Supplemental Table S1** Composition of nutrient solution (manufacturer specification) used in the nutrient film technique system in both greenhouse (LR1) and indoor farming (LR2).

| Nutrient solution | |
| --- | --- |
| NH_4_NO_3_ [mmol/L] | 0.6 |
| Ca(NO3)2 [g/L] | 1.04 |
| KNO_3_ [g/L] | 0.81 |
| Iron chelate [ppm] | 8 |
| KH_2_PO_4_ [g/L] | 0.31 |
| MnSO_4_ [mg/L] | 2.5 |
| MgSO_4_ [g/L] | 0.54 |
| Na_2_[B_4_O_5_(OH)_4_]·8H_2_O [mg/L] | 3.6 |
| CuSO4 [mg/L] | 0.2 |
| Na_2_MoO_4_ [mg/L] | 0.1 |
| ZnSO_4_ [mg/L] | 0.4 |
| pH | 6.2 |

**Supplemental Table S2** Chloride concentrations and water content in the soil of indoor farming (LR2) samples at the end of the experimental period. Means ± SEM; n = 8 from two independent experiments.

|  |  | *Cochlearia officinalis* | | |  | *Atriplex hortensis* | | |  | *Salicornia europaea* | | |
| --- | --- | --- | --- | --- | --- | --- | --- | --- | --- | --- | --- | --- |
| Chloride [mg g^-1^ DW] | 0 | 0.08 | ± | 0.01 |  | 0.10 | ± | 0.01 |  | 0.09 | ± | 0.01 |
|  | 50 | 3.21 | ± | 0.70 |  | 4.41 | ± | 0.54 |  | 2.58 | ± | 0.26 |
|  | 200 | 6.68 | ± | 0.96 |  | 7.47 | ± | 0.48 |  | 7.02 | ± | 0.71 |
|  | 600 | 18.36 | ± | 2.64 |  | 22.66 | ± | 3.35 |  | 16.03 | ± | 1.96 |
| Water content [%] | 0 | 18.42 | ± | 0.78 |  | 19.88 | ± | 0.70 |  | 22.17 | ± | 0.63 |
|  | 50 | 17.75 | ± | 1.75 |  | 19.53 | ± | 0.89 |  | 20.05 | ± | 1.48 |
|  | 200 | 18.10 | ± | 0.72 |  | 19.13 | ± | 1.41 |  | 17.83 | ± | 1.04 |
|  | 600 | 18.65 | ± | 0.79 |  | 19.54 | ± | 0.57 |  | 18.08 | ± | 0.75 |

**Supplemental Table S3** dF, F and p values of Two Way ANOVA’s.

|  |  |  | *Cochlearia officinalis* | | | *Atriplex hortensis* | | | *Salicornia europaea* | | |
| --- | --- | --- | --- | --- | --- | --- | --- | --- | --- | --- | --- |
|  |  |  | DF | F | p | DF | F | p | DF | F | p |
|  | Fresh weight (g) | Interaction | 3 | 26.73 | <0.001 | 3 | 16.78 | <0.001 | 3 | 35.22 | <0.001 |
|  |  | Light regime | 1 | 37.56 | <0.001 | 1 | 95.34 | <0.001 | 1 | 207.50 | <0.001 |
|  |  | Salt treatment | 3 | 206.20 | <0.001 | 3 | 38.70 | <0.001 | 3 | 40.50 | <0.001 |
|  | Fresh weight (FW) (%) | Interaction | 3 | 4.31 | 0.006 | 3 | 0.261 | 0.853 | 3 | 24.60 | <0.001 |
|  |  | Light regime | 1 | 26.17 | <0.001 | 1 | 1.57 | 0.212 | 1 | 73.75 | <0.001 |
|  |  | Salt treatment | 3 | 192.21 | <0.001 | 3 | 70.25 | <0.001 | 3 | 43.04 | <0.001 |
|  | Dry weight (DW) (%) | Interaction | 3 | 3.36 | 0.025 | 3 | 4.88 | 0.005 | 3 | 1.76 | 0.167 |
|  |  | Light regime | 1 | 5.98 | 0.02 | 1 | 11.69 | 0.00 | 1 | 0.52 | 0.476 |
|  |  | Salt treatment | 3 | 81.37 | <0.001 | 3 | 19.95 | <0.001 | 3 | 3.96 | 0.013 |
| Per DW | Chloride | Interaction | 3 | 11.02 | <0.001 | 3 | 28.80 | <0.001 | 3 | 0.50 | 0.684 |
|  |  | Light regime | 1 | 29.76 | <0.001 | 1 | 432.68 | <0.001 | 1 | 2.09 | 0.154 |
|  |  | Salt treatment | 3 | 63.82 | <0.001 | 3 |  | <0.001 | 3 | 148.99 | <0.001 |
|  | ABA | Interaction | 3 | 4.84 | 0.005 | 3 | 3.60 | 0.02 | 3 | 1.93 | 0.137 |
|  |  | Light regime | 1 | 0.35 | 0.555 | 1 | 2.01 | 0.16 | 1 | 2.92 | 0.093 |
|  |  | Salt treatment | 3 | 6.81 | 0.001 | 3 | 3.23 | 0.03 | 3 | 27.96 | <0.001 |
|  | Chlorophyll *a* | Interaction | 3 | 16.12 | <0.001 | 3 | 26.10 | <0.001 | 3 | 12.39 | <0.001 |
|  |  | Light regime | 1 | 274.44 | <0.001 | 1 | 13.50 | <0.001 | 1 | 68.70 | <0.001 |
|  |  | Salt treatment | 3 | 119.82 | <0.001 | 3 | 179.07 | <0.001 | 3 | 23.93 | <0.001 |
|  | Chlorophyll *b* | Interaction | 3 | 18.18 | <0.001 | 3 | 7.60 | <0.001 | 3 | 4.73 | 0.006 |
|  |  | Light regime | 1 | 321.90 | <0.001 | 1 | 16.34 | <0.001 | 1 | 18.93 | <0.001 |
|  |  | Salt treatment | 3 | 96.72 | <0.001 | 3 | 65.66 | <0.001 | 3 | 7.62 | <0.001 |
|  | Lutein | Interaction | 3 | 6.38 | <0.001 | 3 | 20.17 | <0.001 | 3 | 19.49 | <0.001 |
|  |  | Light regime | 1 | 2.38 | <0.001 | 1 | 41.19 | <0.001 | 1 | 92.27 | <0.001 |
|  |  | Salt treatment | 3 | 152.12 | <0.001 | 3 | 310.97 | <0.001 | 3 | 75.53 | <0.001 |
|  | β-Carotene | Interaction | 3 | 32.54 | <0.001 | 3 | 52.61 | <0.001 | 3 | 28.25 | <0.001 |
|  |  | Light regime | 1 | 43.08 | <0.001 | 1 | 7.66 | 0.008 | 1 | 131.21 | <0.001 |
|  |  | Salt treatment | 3 | 86.77 | <0.001 | 3 | 136.93 | <0.001 | 3 | 65.85 | <0.001 |
|  | Zeaxanthin | Interaction | 3 | 0.73 | 0.54 | 3 | 5.83 | 0.002 | 3 | 17.81 | <0.001 |
|  |  | Light regime | 1 | 0.03 | 0.865 | 1 | 0.00 | 0.991 | 1 | 21.39 | <0.001 |
|  |  | Salt treatment | 3 | 8.11 | <0.001 | 3 | 3.92 | 0.013 | 3 | 5.14 | 0.004 |
|  | Violaxanthin | Interaction | 3 | 12.16 | <0.001 | 3 | 4.10 | 0.011 | 3 | 10.89 | <0.001 |
|  |  | Light regime | 1 | 1.37 | 0.247 | 1 | 102.28 | <0.001 | 1 | 7.28 | 0.01 |
|  |  | Salt treatment | 3 | 41.84 | <0.001 | 3 | 55.64 | <0.001 | 3 | 8.81 | <0.001 |
|  | 9*Z*-Neoxanthin | Interaction | 3 | 5.19 | 0.003 | 3 | 1.51 | 0.221 | 3 | 29.76 | <0.001 |
|  |  | Light regime | 1 | 152.00 | <0.001 | 1 | 65.24 | <0.001 | 1 | 62.53 | <0.001 |
|  |  | Salt treatment | 3 | 71.95 | <0.001 | 3 | 8.73 | <0.001 | 3 | 45.11 | <0.001 |
| Per FW | Chloride | Interaction | 3 | 3 | 0.041 | 3 | 2.31 | 0.087 | 3 | 0.01 | 0.998 |
|  |  | Light regime | 1 | 4 | 0.046 | 1 | 4.58 | 0.037 | 1 | 1.47 | 0.231 |
|  |  | Salt treatment | 3 | 15 | <0.001 | 3 | 23.88 | <0.001 | 3 | 13.43 | <0.001 |
|  | ABA | Interaction | 3 | 5 | 0.003 | 3 | 4 | 0.02 | 3 | 1 | 0.264 |
|  |  | Light regime | 1 | 2 | 0.137 | 1 | 8 | 0.006 | 1 | 0 | 0.899 |
|  |  | Salt treatment | 3 | 13 | <0.001 | 3 | 7 | <0.001 | 3 | 5 | 0.004 |
|  | Chlorophyll *a* | Interaction | 3 | 32.62 | <0.001 | 3.00 | 3.37 | 0.025 | 3 | 2.62 | 0.061 |
|  |  | Light regime | 1 | 11.47 | <0.001 | 1.00 | 0.01 | 0.922 | 1 | 17.46 | <0.001 |
|  |  | Salt treatment | 3 | 11.09 | <0.001 | 3.00 | 4.10 | 0.011 | 3 | 8.17 | <0.001 |
|  | Chlorophyll *b* | Interaction | 3 | 26.67 | <0.001 | 3.00 | 6.48 | <0.001 | 3 | 1.64 | 0.192 |
|  |  | Light regime | 1 | 75.43 | <0.001 | 1.00 | 0.00 | 0.957 | 1 | 1.32 | 0.256 |
|  |  | Salt treatment | 3 | 13.55 | <0.001 | 3.00 | 2.31 | 0.087 | 3 | 1.38 | 0.26 |
|  | Lutein | Interaction | 3 | 5.98 | 0.001 | 3.00 | 2.59 | 0.062 | 3 | 2 | 0.212 |
|  |  | Light regime | 1 | 32.69 | <0.001 | 1.00 | 0.08 | 0.775 | 1 | 6 | 0.017 |
|  |  | Salt treatment | 3 | 2.44 | 0.07 | 3.00 | 5.41 | 0.003 | 3 | 14 | <0.001 |
|  | β-Carotene | Interaction | 3 | 23.10 | <0.001 | 3.00 | 3.80 | 0.015 | 3 | 3 | 0.046 |
|  |  | Light regime | 1 | 29.29 | <0.001 | 1.00 | 0.52 | 0.473 | 1 | 9 | 0.004 |
|  |  | Salt treatment | 3 | 8.98 | <0.001 | 3.00 | 5.21 | 0.003 | 3 | 11 | <0.001 |
|  | Zeaxanthin | Interaction | 3 | 2.05 | 0.12 | 3.00 | 3.64 | 0.019 | 3 | 12 | <0.001 |
|  |  | Light regime | 1 | 1.79 | 0.19 | 1.00 | 0.23 | 0.636 | 1 | 27 | <0.001 |
|  |  | Salt treatment | 3 | 9.99 | <0.001 | 3.00 | 5.29 | 0.003 | 3 | 5 | 0.003 |
|  | Violaxanthin | Interaction | 3 | 12.10 | <0.001 | 3.00 | 1.48 | 0.231 | 3 | 9 | <0.001 |
|  |  | Light regime | 1 | 1.96 | 0.17 | 1.00 | 2.72 | 0.105 | 1 | 1 | 0.46 |
|  |  | Salt treatment | 3 | 1.10 | 0.36 | 3.00 | 2.57 | 0.064 | 3 | 7 | <0.001 |
|  | 9*Z*-Neoxanthin | Interaction | 3 | 14.45 | <0.001 | 3.00 | 2.07 | 0.115 | 3 | 5 | 0.003 |
|  |  | Light regime | 1 | 58.75 | <0.001 | 1.00 | 3.40 | 0.071 | 1 | 1 | 0.373 |
|  |  | Salt treatment | 3 | 2.98 | 0.04 | 3.00 | 2.12 | 0.108 | 3 | 14 | <0.001 |

**Supplemental Table S4** Fresh and dry mass of leaves of three pooled plants of 6 to 9 week-old plants in light regime 1 (LR1, greenhouse) and light regime 2 (LR2, indoor farming). n = 8 pools of 3 individual plants each from two independent experiments.

|  |  | Salt treatment [mM NaCl] | Fresh mass [g] | | |  | Dry mass [g] | | |
| --- | --- | --- | --- | --- | --- | --- | --- | --- | --- |
| *Cochlearia officinalis* | Light regime 1 | 0 | 22.91 | ± | 0.82 |  | 1.83 | ± | 0.07 |
|  |  | 50 | 20.64 | ± | 2.01 |  | 0.96 | ± | 0.15 |
|  |  | 200 | 14.37 | ± | 0.85 |  | 0.81 | ± | 0.09 |
|  |  | 600 | 2.22 | ± | 0.21 |  | 0.37 | ± | 0.18 |
|  |  |  |  |  |  |  |  |  |  |
|  | Light regime 2 | 0 | 43.93 | ± | 2.86 |  | 3.55 | ± | 0.35 |
|  |  | 50 | 36.55 | ± | 3.88 |  | 1.01 | ± | 0.38 |
|  |  | 200 | 14.37 | ± | 1.49 |  | 0.47 | ± | 0.22 |
|  |  | 600 | 0.77 | ± | 0.08 |  | 0.09 | ± | 0.04 |
| *Atriplex hortensis* | Light regime 1 | 0 | 17.56 | ± | 1.45 |  | 0.99 | ± | 0.08 |
|  |  | 50 | 21.06 | ± | 1.23 |  | 1.39 | ± | 0.10 |
|  |  | 200 | 10.92 | ± | 0.81 |  | 1.55 | ± | 0.48 |
|  |  | 600 | 1.66 | ± | 0.21 |  | 0.27 | ± | 0.03 |
|  |  |  |  |  |  |  |  |  |  |
|  | Light regime 2 | 0 | 123.49 | ± | 12.36 |  | 11.46 | ± | 1.13 |
|  |  | 50 | 98.28 | ± | 16.15 |  | 10.23 | ± | 1.47 |
|  |  | 200 | 50.08 | ± | 5.29 |  | 6.48 | ± | 0.26 |
|  |  | 600 | 3.80 | ± | 0.52 |  | 1.21 | ± | 0.14 |
| *Salicornia europaea* | Light regime 1 | 0 | 0.41 | ± | 0.07 |  | 0.03 | ± | 0.01 |
|  |  | 50 | 0.70 | ± | 0.10 |  | 0.04 | ± | 0.01 |
|  |  | 200 | 0.84 | ± | 0.09 |  | 0.05 | ± | 0.01 |
|  |  | 600 | 0.56 | ± | 0.56 |  | 0.04 | ± | 0.04 |
|  |  |  |  |  |  |  |  |  |  |
|  | Light regime 2 | 0 | 1.76 | ± | 0.12 |  | 0.09 | ± | 0.02 |
|  |  | 50 | 8.34 | ± | 0.84 |  | 0.41 | ± | 0.06 |
|  |  | 200 | 10.45 | ± | 0.60 |  | 0.62 | ± | 0.05 |
|  |  | 600 | 2.24 | ± | 0.25 |  | 0.21 | ± | 0.02 |

**Supplemental Table S5** Content of chlorophylls on fresh mass basis in leaves of 6 to 9 week-old plants. Means ± SEM of n = 8 pools of 3 individual plants each from two independent experiments. Small letters indicate significant differences between salt treatments in light regime 1 (LR1, greenhouse) in alphabetic order from highest to lowest; capital letters indicate significant differences between salt treatments in light regime 2 (LR2, indoor farming) in alphabetic order from highest to lowest, asterisks indicate significant differences between LR11 and LR2 in-between one salt treatment, interaction shows significant different interaction between salt treatments and light regimes, Two-Way ANOVA, followed by *post hoc* Bonferroni Test (*p* ≤ 0.05)(* ≤ 0.05, ** ≤ 0.01, *** ≤ 0.001); n = 8 pools of 3 individual plants each. ns, not significant.

|  |  | Salt treatment [mM NaCl] | Chlorophyll *a* [ng mg^-1^ FM] | | | | | Chlorophyll *b*  [ng mg^-1^ FM] | | | | |
| --- | --- | --- | --- | --- | --- | --- | --- | --- | --- | --- | --- | --- |
| *Cochlearia officinalis* | Light regime 1 | 0 | 685.90 | ± | 45.03 | B |  | 224.45 | ± | 12.68 | B |  |
|  |  | 50 | 761.33 | ± | 7.35 | B |  | 251.51 | ± | 2.79 | B |  |
|  |  | 200 | 846.84 | ± | 27.47 | B | *** | 243.49 | ± | 45.88 | B | * |
|  |  | 600 | 1516.45 | ± | 106.65 | A | *** | 623.95 | ± | 87.44 | A | *** |
|  |  |  |  |  |  |  |  |  |  |  |  |  |
|  | Light regime 2 | 0 | 569.19 | ± | 21.91 | AB |  | 182.16 | ± | 7.39 | ns |  |
|  |  | 50 | 621.00 | ± | 25.53 | A |  | 199.34 | ± | 8.87 | ns |  |
|  |  | 200 | 482.76 | ± | 57.81 | AB |  | 160.36 | ± | 20.61 | ns |  |
|  |  | 600 | 363.86 | ± | 108.99 | AB |  | 129.42 | ± | 38.44 | ns |  |
|  | **Interaction** light regime x salt treatment | | | | | *** |  |  |  |  | *** |  |
| *Atriplex hortensis* | Light regime 1 | 0 | 179.11 | ± | 8.84 | b |  | 40.70 | ± | 0.74 | ns |  |
|  |  | 50 | 217.29 | ± | 7.65 | b |  | 45.34 | ± | 1.74 | ns |  |
|  |  | 200 | 335.91 | ± | 92.02 | a |  | 66.61 | ± | 25.40 | ns |  |
|  |  | 600 | 281.37 | ± | 25.36 | ab |  | 67.22 | ± | 4.95 | ns | *** |
|  |  |  |  |  |  |  |  |  |  |  |  |  |
|  | Light regime 2 | 0 | 336.00 | ± | 8.22 | ns |  | 65.63 | ± | 1.16 | A |  |
|  |  | 50 | 357.12 | ± | 16.83 | ns |  | 66.35 | ± | 2.39 | A |  |
|  |  | 200 | 356.99 | ± | 74.28 | ns |  | 69.92 | ± | 14.37 | A |  |
|  |  | 600 | 91.83 | ± | 21.43 | ns |  | 19.43 | ± | 4.76 | B |  |
|  |  |  |  |  |  |  |  |  |  |  |  |  |
|  | **Interaction** light regime x salt treatment | | | | |  | * |  |  |  |  | *** |
| *Salicornia europaea* | Light regime 1 | 0 | 35.49 | ± | 18.32 | ns |  | 33.77 | ± | 12.74 | ns |  |
|  |  | 50 | 45.13 | ± | 16.60 | ns |  | 31.24 | ± | 3.97 | ns |  |
|  |  | 200 | 90.18 | ± | 9.05 | ns |  | 30.71 | ± | 5.85 | ns |  |
|  |  | 600 | 50.23 | ± | 13.58 | ns |  | 22.47 | ± | 5.73 | ns |  |
|  |  |  |  |  |  |  |  |  |  |  |  |  |
|  | Light regime 2 | 0 | 33.94 | ± | 14.79 | b |  | 20.37 | ± | 7.46 | ns |  |
|  |  | 50 | 135.88 | ± | 13.28 | a | *** | 43.65 | ± | 4.75 | ns |  |
|  |  | 200 | 147.87 | ± | 15.70 | a | * | 44.02 | ± | 5.10 | ns |  |
|  |  | 600 | 105.30 | ± | 23.49 | a | * | 32.90 | ± | 7.28 | ns |  |
|  |  |  |  |  |  |  |  |  |  |  |  |  |
|  | **Interaction** light regime x salt treatment | | | | |  | ns |  |  |  |  | ns |

**Supplemental Table S6** Content of carotenoids on fresh mass basis in leaves of 6 to 9 week-old plants. Means ± SEM of n = 8 pools of 3 individual plants each from two independent experiments. Small letters indicate significant differences between salt treatments in light regime 1 (LR1, greenhouse) in alphabetic order from highest to lowest; capital letters indicate significant differences between salt treatments in light regime 2 (LR2, indoor farming) in alphabetic order from highest to lowest, asterisks indicate significant differences between LR1 and LR2 in-between one salt treatment, interaction shows significant different interaction between salt treatments and light regimes, Two-Way ANOVA, followed by *post hoc* Bonferroni Test (*p* ≤ 0.05)(* ≤ 0.05, ** ≤ 0.01, *** ≤ 0.001); n = 8 pools of 3 individual plants each. ns, not significant.

|  |  | Salt treatment [mM NaCl] | Lutein [ng mg-1 FM] | | | | | β-Carotene [ng mg-1 FM] | | | | | Zeaxanthin [ng mg-1 FM] | | | | | *all-trans*-Violaxanthin (isomer 1) [ng mg-1 FM] | | | | | *9Z*-Neoxanthin (isomer 1) [ng mg-1 FM] | | | | | |
| --- | --- | --- | --- | --- | --- | --- | --- | --- | --- | --- | --- | --- | --- | --- | --- | --- | --- | --- | --- | --- | --- | --- | --- | --- | --- | --- | --- | --- |
| *Cochlearia officinalis* | Light  regime 1 | 0 | 81.16 | ± | 3.88 | b |  | 24.00 | ± | 1.70 | c |  | 1.14 | ± | 0.07 | b |  | 6.11 | ± | 0.24 | b |  | | 19.58 | ± | 1.37 | b |  |
|  |  | 50 | 92.24 | ± | 1.38 | b |  | 33.99 | ± | 0.99 | bc |  | 1.68 | ± | 0.24 | b |  | 6.69 | ± | 0.40 | ab |  | | 22.94 | ± | 0.61 | b |  |
|  |  | 200 | 102.92 | ± | 4.09 | b | ** | 44.00 | ± | 1.36 | b | * | 2.36 | ± | 0.42 | b |  | 8.88 | ± | 1.20 | ab |  | | 23.08 | ± | 1.08 | b | ** |
|  |  | 600 | 157.54 | ± | 28.59 | a | *** | 77.19 | ± | 9.24 | a | *** | 8.33 | ± | 2.37 | a |  | 10.12 | ± | 1.95 | a | *** | | 41.97 | ± | 6.62 | a | *** |
|  |  |  |  |  |  |  |  |  |  |  |  |  |  |  |  |  |  |  |  |  |  |  | |  |  |  |  |  |
|  | Light  regime 2 | 0 | 64.42 | ± | 2.04 | ns |  | 28.30 | ± | 1.18 | AB |  | 0.80 | ± | 0.05 | ns |  | 10.42 | ± | 1.03 | a | ** | | 15.86 | ± | 0.75 | ns |  |
|  |  | 50 | 70.85 | ± | 2.34 | ns |  | 37.01 | ± | 1.22 | A |  | 1.62 | ± | 0.14 | ns |  | 8.15 | ± | 0.67 | a |  | | 16.70 | ± | 0.76 | ns |  |
|  |  | 200 | 55.40 | ± | 6.24 | ns |  | 29.91 | ± | 3.56 | AB |  | 2.94 | ± | 0.42 | ns |  | 6.46 | ± | 0.84 | ab |  | | 10.87 | ± | 1.44 | ns |  |
|  |  | 600 | 49.72 | ± | 14.94 | ns |  | 19.43 | ± | 5.89 | B |  | 4.21 | ± | 1.64 | ns |  | 2.73 | ± | 0.63 | b |  | | 6.53 | ± | 1.76 | ns |  |
|  |  |  |  |  |  |  |  |  |  |  |  |  |  |  |  |  |  |  |  |  |  |  | |  |  |  |  |  |
|  | **Interaction** light regime x salt treatment | | | | |  | *** |  |  |  |  | *** |  |  |  |  | ns |  |  |  |  | *** | |  |  |  |  | *** |
| *Atriplex hortensis* | Light regime 1 | 0 | 16.49 | ± | 0.30 | ab |  | 8.83 | ± | 0.38 | b |  | 0.38 | ± | 0.05 | b |  | 4.09 | ± | 0.21 | ns |  | | 4.13 | ± | 0.11 | ns |  |
|  |  | 50 | 20.03 | ± | 0.75 | ab |  | 12.29 | ± | 0.49 | b |  | 0.86 | ± | 0.25 | b |  | 5.63 | ± | 0.25 | ns |  | | 4.76 | ± | 0.18 | ns |  |
|  |  | 200 | 44.21 | ± | 15.26 | a |  | 33.96 | ± | 12.10 | a |  | 5.15 | ± | 1.95 | a | ** | 10.28 | ± | 4.09 | ns |  | | 10.96 | ± | 3.81 | ns |  |
|  |  | 600 | 18.93 | ± | 1.66 | b |  | 18.41 | ± | 2.25 | ab |  | 1.49 | ± | 0.42 | b |  | 4.56 | ± | 0.57 | ns |  | | 6.28 | ± | 0.53 | ns |  |
|  |  |  |  |  |  |  |  |  |  |  |  |  |  |  |  |  |  |  |  |  |  |  | |  |  |  |  |  |
|  | Light  regime 2 | 0 | 28.65 | ± | 0.63 | AB |  | 23.05 | ± | 0.47 | AB |  | 0.99 | ± | 0.10 | ns |  | 3.01 | ± | 0.68 | ns |  | | 5.32 | ± | 0.79 | ns |  |
|  |  | 50 | 29.19 | ± | 1.34 | AB |  | 26.80 | ± | 1.20 | A | ** | 1.02 | ± | 0.33 | ns |  | 6.40 | ± | 0.79 | ns |  | | 4.18 | ± | 1.14 | ns |  |
|  |  | 200 | 30.04 | ± | 5.75 | A |  | 27.76 | ± | 5.62 | A |  | 1.80 | ± | 0.44 | ns |  | 4.54 | ± | 0.97 | ns |  | | 4.79 | ± | 0.86 | ns |  |
|  |  | 600 | 6.87 | ± | 1.92 | B |  | 6.13 | ± | 1.47 | B |  | 2.99 | ± | 0.45 | ns |  | 2.96 | ± | 0.51 | ns |  | | 3.70 | ± | 1.02 | ns |  |
|  |  |  |  |  |  |  |  |  |  |  |  |  |  |  |  |  |  |  |  |  |  |  | |  |  |  |  |  |
|  | **Interaction** light regime x salt treatment | | | | |  | ns |  |  |  |  | * |  |  |  |  | * |  |  |  |  | ns | |  |  |  |  | ns |
| *Salicornia europaea* | Light  regime 1 | 0 | 3.23 | ± | 1.03 | b |  | 1.06 | ± | 0.08 | ns |  | 1.71 | ± | 0.05 | a | *** | 1.38 | ± | 0.20 | ns |  | | 1.43 | ± | 0.15 | ns |  |
|  |  | 50 | 7.30 | ± | 1.87 | ab |  | 1.92 | ± | 0.70 | ns |  | 0.36 | ± | 0.04 | b |  | 1.29 | ± | 0.27 | ns |  | | 1.46 | ± | 0.34 | ns |  |
|  |  | 200 | 15.39 | ± | 2.07 | a |  | 4.91 | ± | 0.81 | ns |  | 0.34 | ± | 0.11 | b |  | 2.22 | ± | 0.41 | ns |  | | 3.36 | ± | 0.32 | ns | * |
|  |  | 600 | 8.36 | ± | 3.13 | b |  | 1.75 | ± | 1.20 | ns |  | 0.49 | ± | 0.05 | b |  | 1.33 | ± | 0.06 | ns |  | | 2.02 | ± | 0.37 | ns |  |
|  |  |  |  |  |  |  |  |  |  |  |  |  |  |  |  |  |  |  |  |  |  |  | |  |  |  |  |  |
|  | Light  regime 2 | 0 | 2.58 | ± | 1.38 | b |  | 0.27 | ± | 0.12 | b |  | 0.05 | ± | 0.36 | ns |  | 0.65 | ± | 0.46 | B |  | | 0.60 | ± | 0.24 | c |  |
|  |  | 50 | 15.80 | ± | 1.03 | a | *** | 6.69 | ± | 0.61 | a | *** | 0.27 | ± | 0.11 | ns |  | 2.83 | ± | 0.13 | A | *** | | 3.32 | ± | 0.10 | ab | *** |
|  |  | 200 | 18.75 | ± | 2.98 | a |  | 7.96 | ± | 0.49 | a | * | 0.37 | ± | 0.07 | ns |  | 1.48 | ± | 0.15 | B |  | | 3.60 | ± | 0.56 | a |  |
|  |  | 600 | 13.07 | ± | 2.10 | a |  | 5.09 | ± | 0.67 | a |  | 0.33 | ± | 0.18 | ns |  | 0.88 | ± | 0.22 | B |  | | 2.08 | ± | 0.44 | b |  |
|  |  |  |  |  |  |  |  |  |  |  |  |  |  |  |  |  |  |  |  |  |  |  | |  |  |  |  |  |
|  | **Interaction** light regime x salt treatment | | | | |  | ns |  |  |  |  | * |  |  |  |  | *** |  |  |  |  | *** | |  |  |  |  | ** |

**Supplemental Figures**


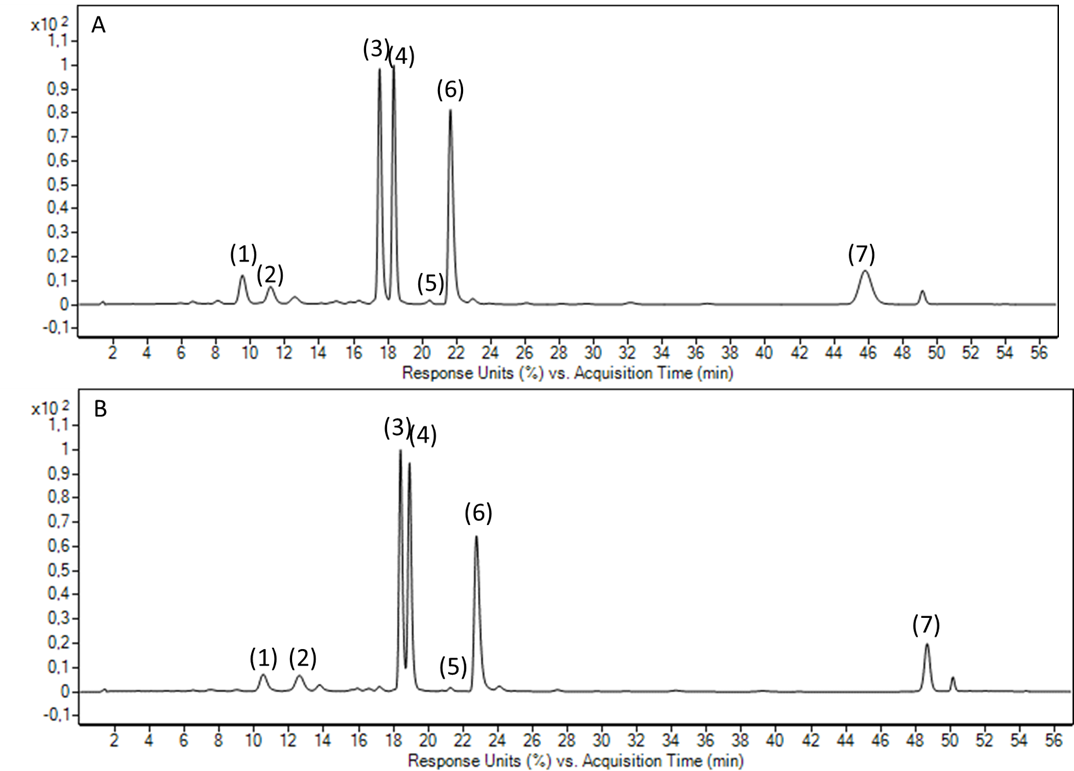


**Supplemental Figure S1** HPLC-DAD chromatogram at 450 nm showing identified chlorophylls and carotenoids exemplary in leaves of *Cochlearia officinalis* grown in (A) greenhouse (LR1) and (B) indoor farming (LR2). (1) *all-trans*-violaxanthin; (2) 9*Z*-neoxanthin; (3) chlorophyll *b*; (4) *all-trans*-lutein; (5) *all-trans*-zeaxanthin; (6) chlorophyll *a*; (7) *all-trans*-β-carotene.


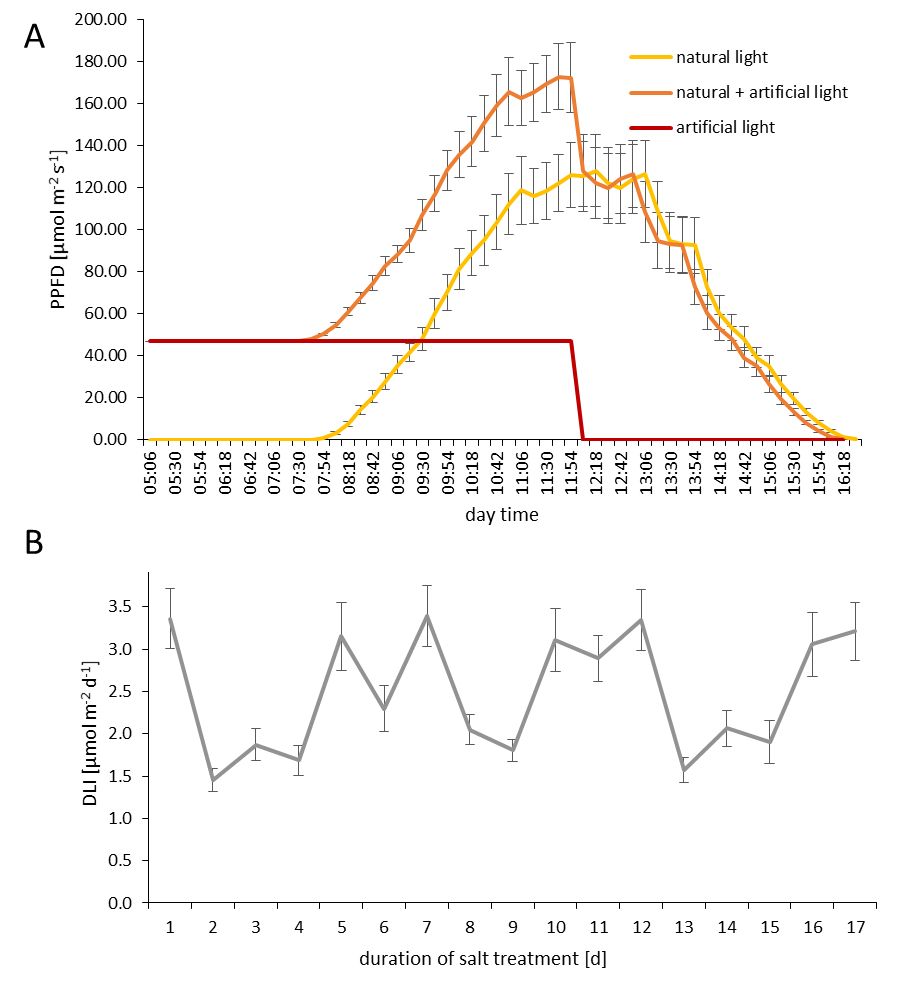


**Supplemental Figure S2** Average light distribution in greenhouse (LR1). (A) Average light intensity over the day of natural and artificial light represented in photosynthetic photon flux density (PPFD). (B) Fluctuations of the daily light integral (DLI) over the salt treatment period. Means ± SEM.

**
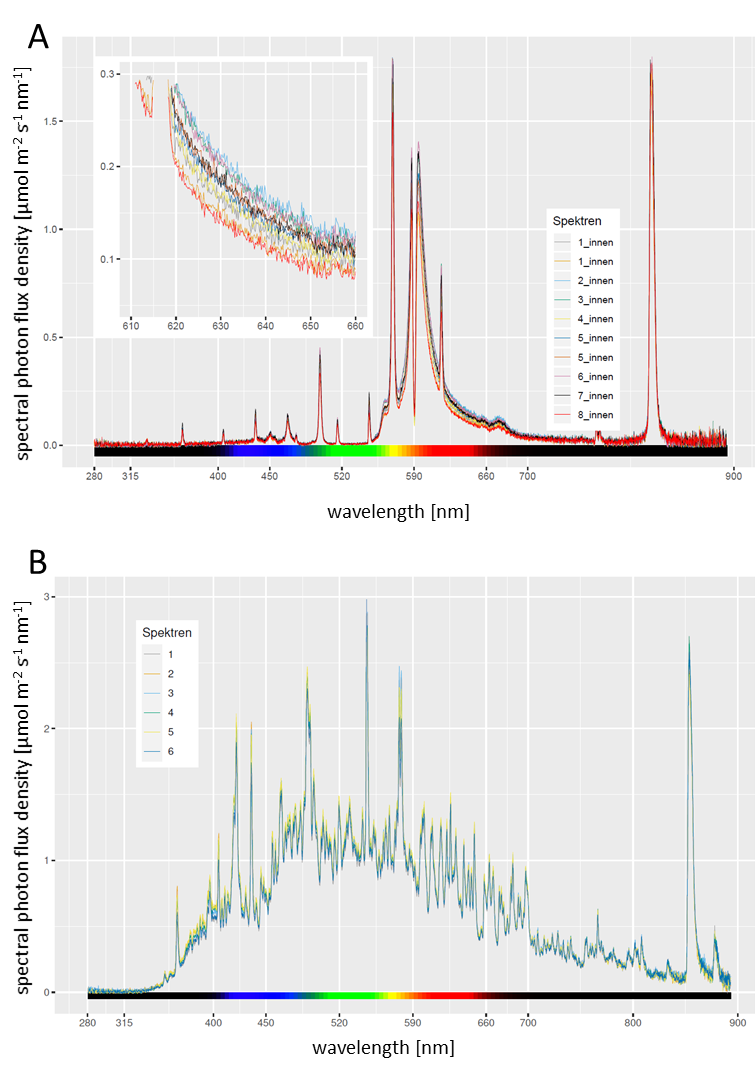
**

**Supplemental Figure S3** Light spectra in (A) greenhouse (LR1) and (B) indoor farming (LR2) measured with a spectrophotometer (Ocean Insight, US).





**Supplemental Figure S4** Sodium chloride concentrations in nutrient solution during the experimental period of 17 days in greenhouse (LR1) and indoor farming (LR2). (A) No salt; (B) 50 mM; (C) 200 mM and (D) 600 mM. LR1-1, replicate experiment 1 (greenhouse); LR1-2, replicate experiment 2 (greenhouse); LR2-1, replicate experiment 1 (indoor farming); LR2-2, replicate experiment 2 (indoor farming).


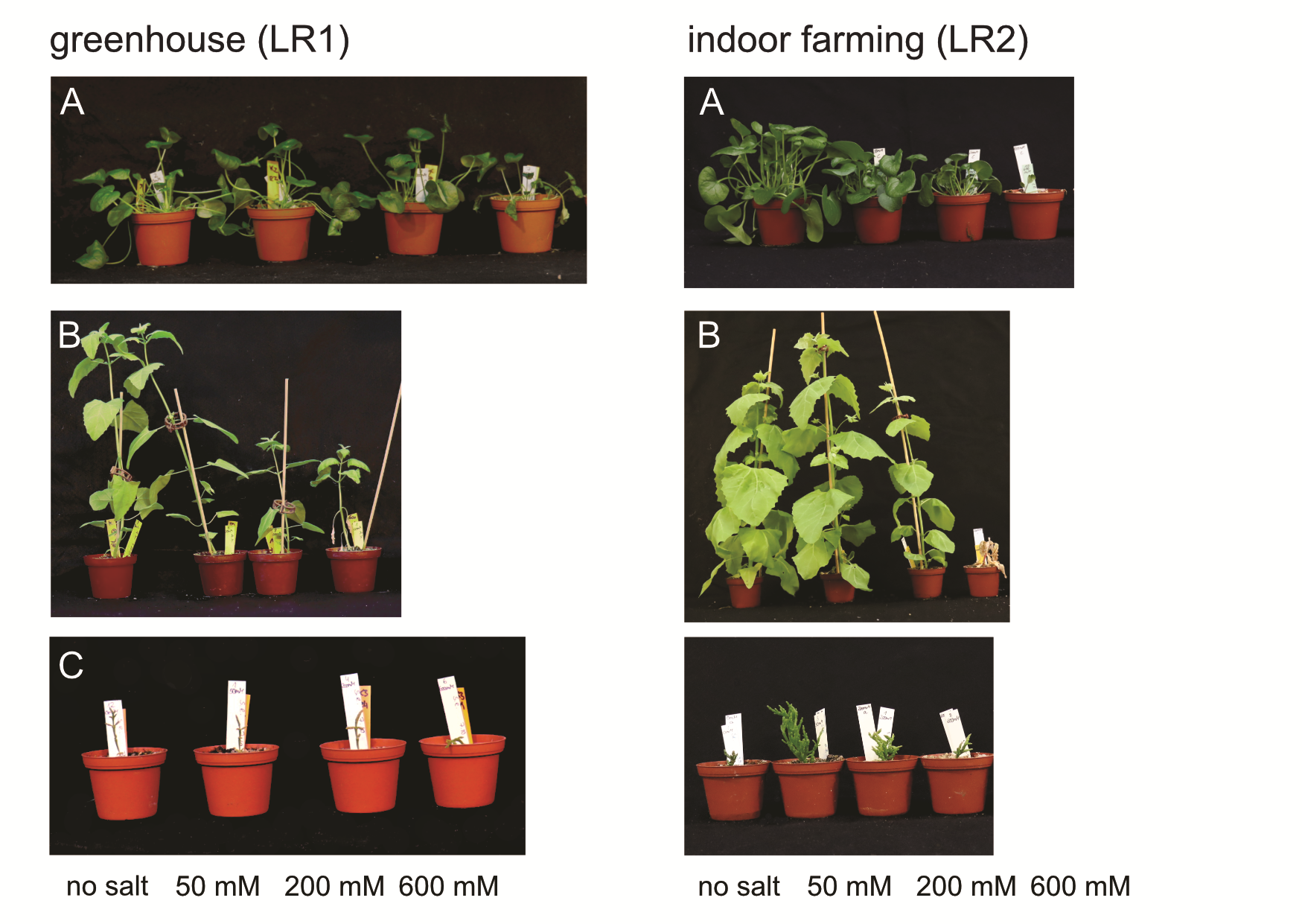


**Supplemental Figure S5** Effect of salt treatment on the phenotypes of (A) *Cochlearia officinalis*; (C) *Atriplex hortensis*; (C) *Salicornia europaea* in greenhouse (LR1) and indoor farming (LR2) 6 or 9 week-old plants depending on plant species, after 3 weeks of salt treatment. Increasing salt treatment (in mM NaCl) from left to right.


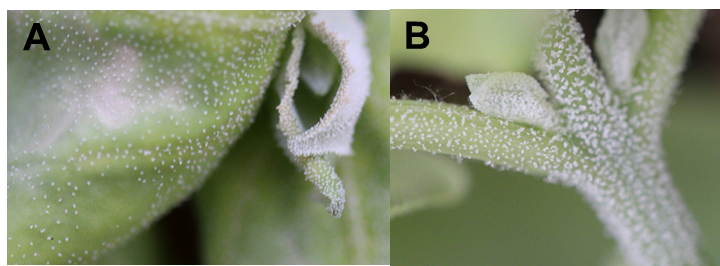


**Supplemental Figure S6** Salt bladders/glands. Magnification (1.4-fold) of (A) leaves and (B) stem of *Atriplex hortensis*; at 600 mM salt treatment in indoor farming (LR2) after 5 days of salt treatment.





**Supplemental Figure S7** Effect of salt treatment and light regime on fresh weight calculated as percent of no salt. (A) *Cochlearia officinalis*; (C) *Atriplex hortensis*; (C) *Salicornia europaea* in greenhouse (LR1) and indoor farming (LR2). Means ± SEM of n = 24. Small letters indicate significant differences between salt treatments in light regime 1 in alphabetic order from highest to lowest; capital letters indicate significant differences between salt treatments in light regime 2 in alphabetic order from highest to lowest, asterisks indicate significant differences between light regime 1 and 2 in-between one salt treatment, interaction shows significant different interaction between salt treatments and light regimes tested by Two-Way ANOVA, followed by post hoc Bonferroni Test (p ≤ 0.05)(* ≤ 0.05, ** ≤ 0.01, *** ≤ 0.001).


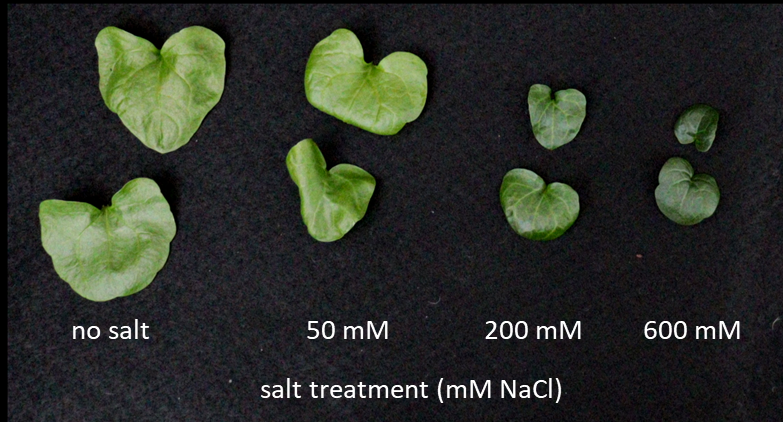


**Supplemental Figure S8** Influence of salt treatment on leaves of *Cochlearia officinalis*. Six week-old plants after 17 days of salt treatment in the greenhouse (LR1).
